# Unexpected cranial sexual dimorphism in the tragulid *Dorcatherium naui* based on material from the middle to late Miocene localities of Eppelsheim and Hammerschmiede (Germany)

**DOI:** 10.1371/journal.pone.0267951

**Published:** 2022-05-18

**Authors:** Josephina Hartung, Madelaine Böhme

**Affiliations:** 1 Department of Geosciences, Eberhard Karls University, Tübingen, Germany; 2 Senckenberg Centre for Human Evolution and Paleoenvironment (HEP), Tübingen, Germany; Griffith University, AUSTRALIA

## Abstract

Tragulids, chevrotains or mouse deer, were common faunal elements during the Miocene. During that time, *Dorcatherium* was the most abundant genus, with *D*. *naui* being the first described species. Besides their abundance, until recently only very limited cranial material was available for investigation. Here we present a redescription of the first complete skull of *D*. *naui* from the middle to late Miocene locality of Eppelsheim, Germany, based on micro-computed tomography. Furthermore, we present a description and comparison of two additional, new skulls of *D*. *naui* from the late Miocene hominid locality Hammerschmiede, Germany. Within *Dorcatherium*, so far, only three other complete skulls are known, all belonging to *D*. *crassum*. A comparison between the three skulls of *D*. *naui* and the already known skulls of *D*. *crassum* shows that these two species differ in morphological features of the skull, such as laterally facing orbitae, separation of supraorbital foramen from supraorbital groove by a bony bridge, well-developed parietal plateau, prominent nuchal tubercle, less-developed nuchal crests, and the presence of an occipital crest. Moreover, two different osteological morphotypes are present in the skulls of *D*. *naui* that can be interpreted as a previously unknown sexual dimorphism. Very similar features are observed in *D*. *crassum*, which can be likewise related to the same dimorphism. However, males of *D*. *naui* differ from males of *D*. *crassum* by the presence of frontoparietal bulges, which were probably used for sexual display and during male-male combats in males of *D*. *naui*. For the first time, sexual dimorphism in *Dorcatherium* is described based on skull characteristics, which are, so far, unknown from any other fossil or extant tragulid.

## Introduction

Tragulidae (mouse-deer or chevrotains) are a group of ruminants and the sister group to Pecora. They contain one of the smallest living artiodactyles and survived until today in Asia (*Tragulus* and *Moschiola*) and Africa (*Hyemoschus*) [1]. Tragulidae are characterized by the absence of cranial appendages, bunoselenodont to selenodont dentition, the presence of a Ʃ-structure in the lower molars, and large, saber-like upper canines in males [2]. The precise evolutionary history of the family is poorly known with the exception that it originated in the late Eocene of Asia [2–5], which was recently confirmed by Mennecart et al. [6], who report the oldest true tragulid from the Eocene of China.

Tragulidae show a high species diversification and distribution during the Miocene with *Dorcatherium* being the most diverse tragulid genus during that time. Rössner and Heissig (Fig 7 in [7]) and Aiglstorfer et al. (Fig 1 in [8]) show that *Dorcatherium* reaches its highest diversity in central Europe during the early middle Miocene with four contemporaneous species around 15 Ma.

Despite their diversity and distribution, the ingroup taxonomy of *Dorcatherium* is in need of revision [9], because complete skulls are rare and most taxonomic studies are based on isolated tooth material only. So far, only few skulls of *Dorcatherium* are described and all belong to *Dorcatherium crassum* [10–12] except for one complete skull of *D*. *naui*, described by Kaup [13, 14] from Eppelsheim, Germany. However, no detailed description or illustration of the skull from Eppelsheim was available leading to little attention after its original description and in the recent literature it was only very briefly described [15]. Another skull from Steinheim am Albuch was first assigned to *D*. *naui* [16], but later reassigned to *D*. *guntianum* [7] or *D*. *crassum* [8].

Here we focus our study on the skull morphology of the type species of *Dorcatherium*, *D*. *naui*. The goal of this manuscript is the redescription and comparison of the skull from the type locality of *D*. *naui*, Eppelsheim, Germany together with two new skulls from the late Miocene hominid locality Hammerschmiede. For the first time it is possible to show that despite the characteristic dentition, *D*. *naui* exposes unique features of the skull.

### Geological setting

The Hammerschmiede locality is located in southern Germany (Allgäu, Bavaria) in the Northern Alpine Foreland Basin. The sediments comprise floodplain deposits belonging to the *Upper Series*, the youngest lithostratigraphic part of the Upper Freshwater Molasse [17, 18]. Two fossil bearing levels HAM 5 and HAM 4 represent meandering fluvial channels [19], which contain a rich fauna including bivalves and gastropods [20], fishes, amphibians, reptiles, small mammals, large mammals [21–26], and birds [27, 28]. The locality became famous through the discovery of associated skeletons of the hominid *Danuvius guggenmosi* [22, 23]. Biostratigraphic, as well as magnetostratigraphic age constraints place both levels at the beginning of the late Miocene (Tortonian), with absolute ages of 11.62 Ma for HAM 5 and 11.44 Ma for HAM 4 [19].

The Eppelsheim locality represents fluvial deposits of the Eppelsheim Formation from the Mainz Basin containing a middle to late Miocene mixed faunal association of repeatedly reworked paleo-Rhine sediments [29]. Based on the preservation, the complete skull NHMUK PV OR 40632 of *D*. *naui* from Eppelsheim was probably not reworked. An exact age for this skull is not reported, but it occurs within the biostratigraphic distribution of *D*. *naui* given by Aiglstorfer et al. [8] from the early Sarmatian (~12.5 Ma) to Pannonian (~9 Ma).

## Material and methods

The material used in this study comprises one complete (GPIT/MA/18000-01) skull with an associated mandible (GPIT/MA/18000-53) and one partial skull (GPIT/MA/17690) of *Dorcatherium naui* from Hammerschmiede; both from level HAM 4. Moreover, we describe the μCT scan of NHMUK PV OR 40632, an almost complete skull with attached jaws from Eppelsheim (Germany), that was originally published by Kaup [13, 14], housed at the Natural History Museum of London (NHMUK). A cast (GPIT/MA/03653) of this specimen is also available for study at GPIT. For comparison, we used the three best known and almost complete skulls of *D*. *crassum* from Thierhaupten [10] and Walda 2, Germany [11], as well as from Contres, France [12]. Additionally, extant artiodactyls from GPIT (unnumbered specimens) including *Tragulus* sp. (GPIT/MA/12997), *T*. *napu* (GPIT/MA/12998), *T*. *javanicus* (GPIT/MA/12999), and *Hyemoschus* (FMNH 34294) were used.

The terminology of the dentition follows [30] and the tooth measurements are based on [8, 31] with W being the maximum anterior width and L being the maximum length. The skull terminology follows [32, 33] and the skull measurements of *D*. *crassum* are provided according to Fig 4.3 in [11] except the postfrontal length and length/height of the sagittal crest, which were done using the surface model provided by [11]. In addition, the postfrontal skull length was measured in dorsal view at the midline between the frontoparietal suture and the maximum posterior extension of the skull. Measurements of *D*. *naui* from Hammerschmiede and the cast of the specimen from Eppelsheim were taken with a digital caliper with precision of 0.1 mm. The Natural History Museum of London provided a μCT scan of the skull NHMUK PV OR 40632 for this study. The resolution was equal to 0.095 mm voxelsize.

GPIT/MA/17690 from Hammerschmiede was scanned at the Centre of Visualisaiton, Digitisation, and Replication (VDR) at the Eberhard Karls University of Tübingen, Germany. The scans were acquired using the Nikon XTH 320 Reflection Target with an acceleration voltage of 190 kV. For specimen GPIT/MA/17690, the resolution was set to 177 μA. A total of 4476 images were recorded and a 1 mm copper filter was inserted. Images were processed and reconstructed using VG Studio Max 3.4.1.

## Systematic paleontology

MAMMALIA Linnaeus, 1758

ARTIODACTYLA Owen, 1848

RUMINANTIA Scopoli, 1777

TRAGULIDAE Milne Edwards, 1864

Genus *Dorcatherium* Kaup, 1833

Type species: *Dorcatherium naui* Kaup, 1833

*Dorcatherium naui* Kaup, 1833

### Emended diagnosis

Mid-sized selenodont tragulid with (I) a high degree of selenodonty having a reduced external postmetacristid (*sensu* [30]) in the lower molars and well-developed cristids and flat main cusps (*sensu* [34]), (II) prominent sickle-shaped cones and conids (*sensu* [7]) in combination with (III) the presence of the *Dorcatherium*-platform in the lower molars (*sensu* [35]), (IV) a complex posterior valley of the p4 (*sensu* [8]), (V) lateral facing orbitae, (VI) prominent nuchal tubercle, and (VII) the presence of an occipital crest.

Differs from *D*. *crassum* in: (A) higher degree of selenodonty, (B) strongly reduced postmetacristid, (C) laterally facing orbitae instead of dorsolaterally oriented orbitae, (D) the separation of supraorbital foramen from supraorbital groove by a bony bridge, (E) well-developed parietal plateau, (F) prominent nuchal tubercle, (G) less-developed nuchal crests, and (H) the presence of an occipital crest.

#### Holotype

Mandible with p3–m3 and alveoli of p2 and p1, first described by Kaup [36] and later figured by Kaup (pl. 23, Fig 1-1b in [14]). The holotype is lost, but two casts are available, one at the NHMUK (NHMUK PV M 3714) and the other one at the SNSB-BSPG (SNSB–BSPG 1961 XIX 37).

#### Type locality

Eppelsheim, Germany (middle to late Miocene [29]).

#### Referred material

The complete skull NMHUK PV OR 40632 from Eppelsheim (middle to late Miocene of Germany) (Figs 1 and 2), a complete skull GPIT/MA/18000-01 (Figs 3–6) with associated remains of a mandible (GPIT/MA/18000-53) (Fig 7) found in association to the skull resembling the same wear stage as GPIT/MA/18000-01 and therefore belonging to one individual, as well as a partial skull GPIT/MA/17690 (Figs 8–12), both from the Hammerschmiede locality (late Miocene of Germany) level HAM 4.

**Fig 1:**
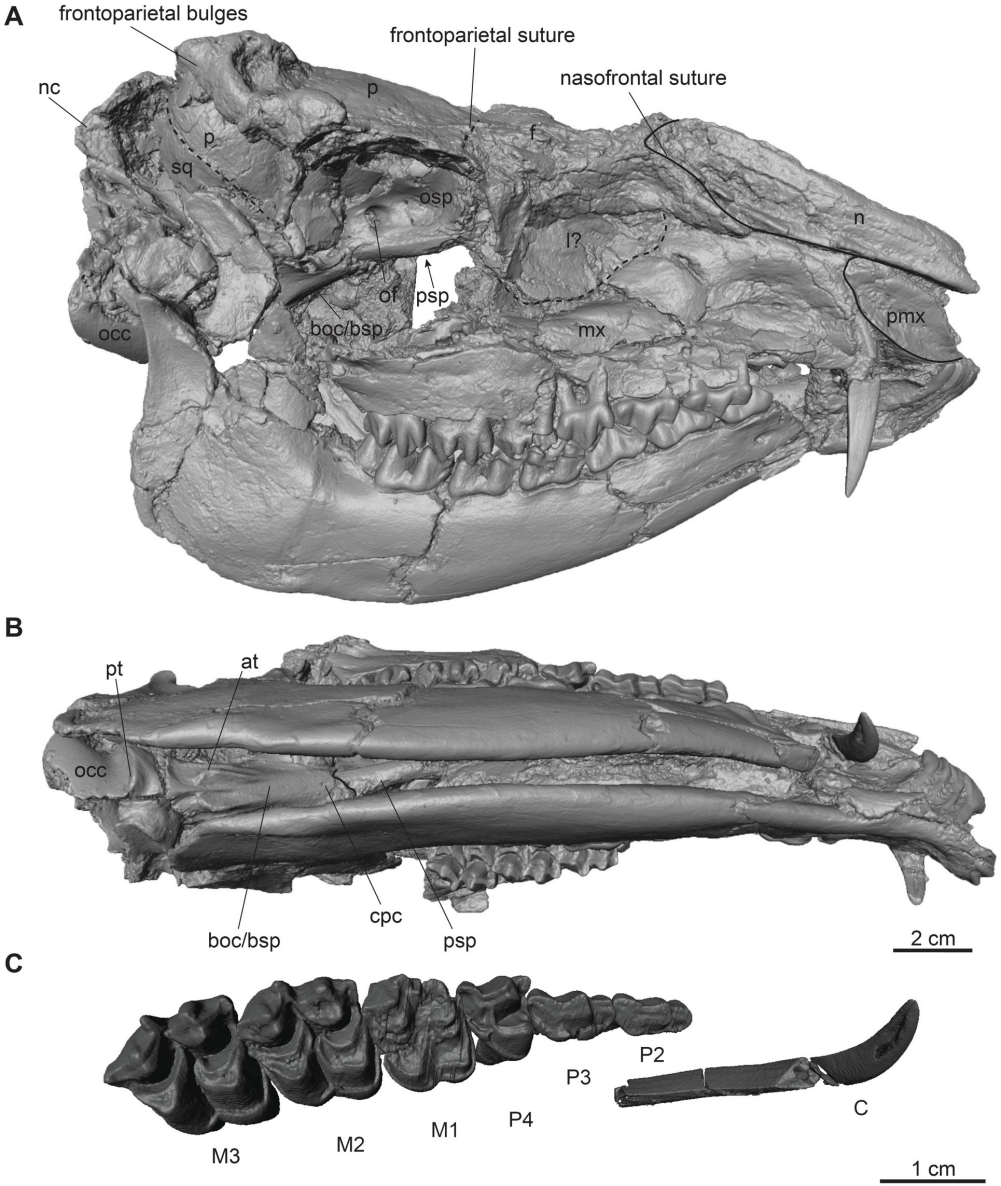
Complete male skull of *Dorcatherium naui* from Eppelsheim, Germany. Surface model of NHMUK PV OR 40632. (A) lateral view, right side, (B) ventral view, (C) close up of right upper tooth row. Abbreviations: at, anterior tuberosities; boc/bsp, basioccipital/basisphenoid; cpc, craniopharyngeal canal; f, frontal; l?, lacrimal; mx, maxillary; n, nasal; nc, nuchal crests; occ, occipital condyle; of, optic foramen; osp, orbitosphenoid; p, parietal; pmx, premaxillary; psp, presphenoid; pt, posterior tuberosities; sq, squamosal.

**Fig 2:**
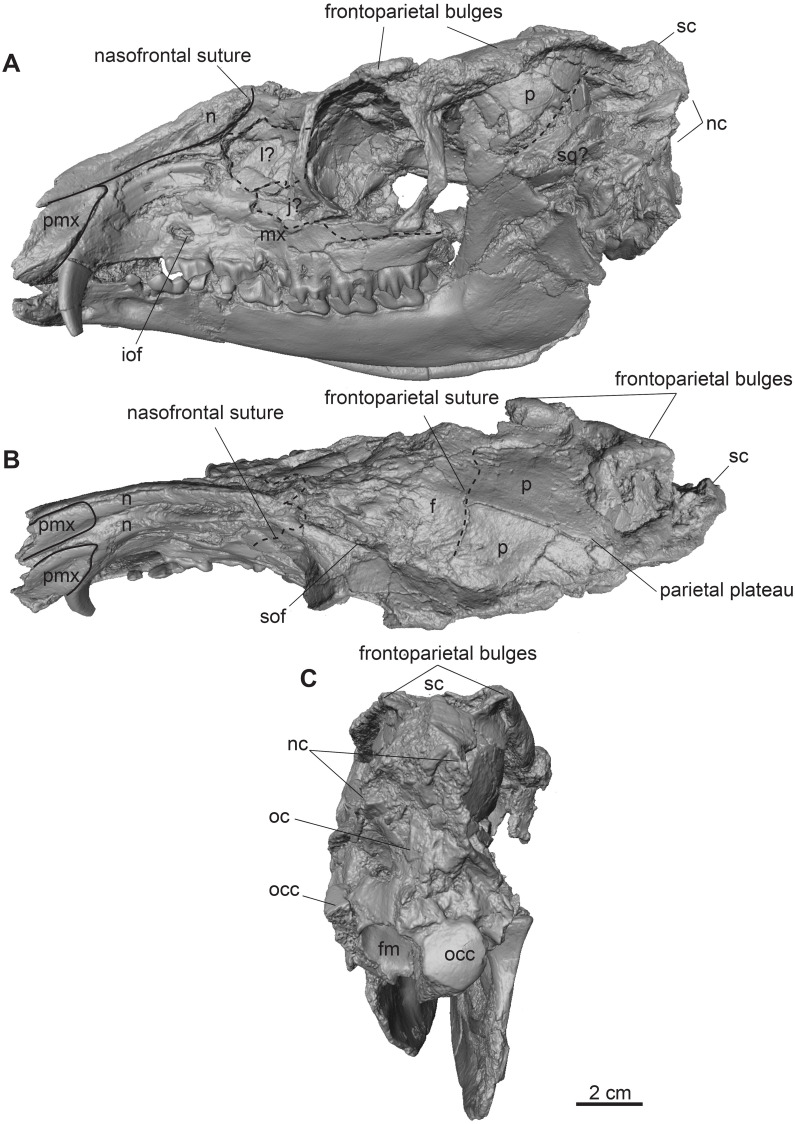
Complete male skull of *Dorcatherium naui* from Eppelsheim, Germany. Surface model of NHMUK PV OR 40632. (A) lateral view, left side, (B) dorsal view, (C) posterior view. Abbreviations: f, frontal; fm, foramen magnum; iof, infraorbital foramen; j?, jugal; l?, lacrimal; mx, maxillary; n, nasal; nc, nuchal crests; oc, occipital crest; occ, occipital condyle; p, parietal; pmx, premaxillary; sc, sagittal crest; sof, supraorbital foramen; sq?, squamosal.

**Fig 3:**
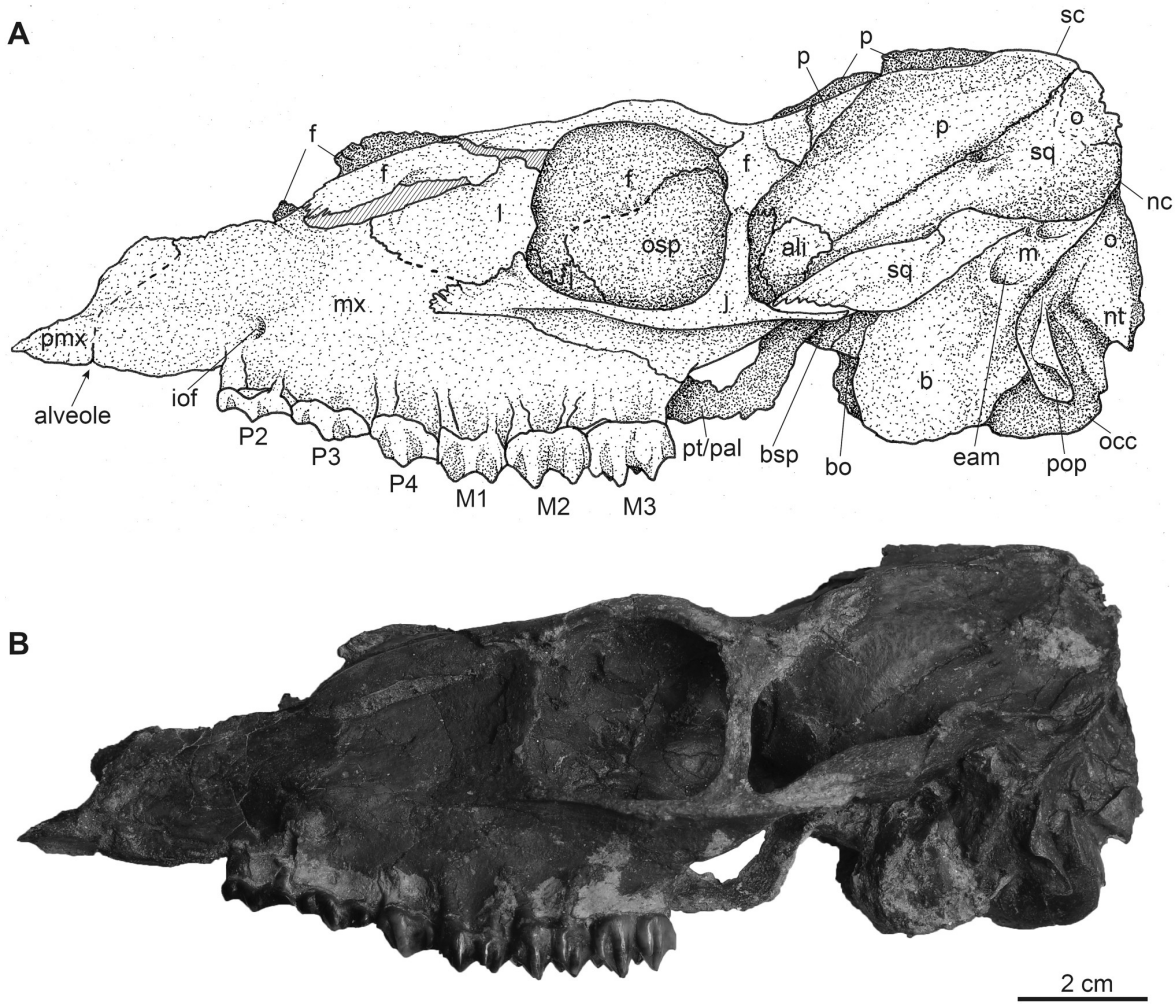
Complete female skull of *Dorcatherium naui* (GPIT/MA/18000-01) in lateral view from the Hammerschmiede locality, Germany (HAM 4). (A) drawing, (B) photograph. Abbreviations: ali, alisphenoid; b, bulla tympanica; bsp, basisphenoid; bo, basioccipital; eam, external auditory meatus; f, frontal; iof, infraorbital foramen; j, jugal; l, lacrimal; mx, maxillary; nc, nuchal crests; nt, nuchal tubercle; o, occipital; occ, occipital condyle; osp, orbitosphenoid; p, parietal; pmx, premaxillary; pop, paroccipital process; pt/pal, pterygoid/palatine; sc, sagittal crest; sq, squamosal.

**Fig 4:**
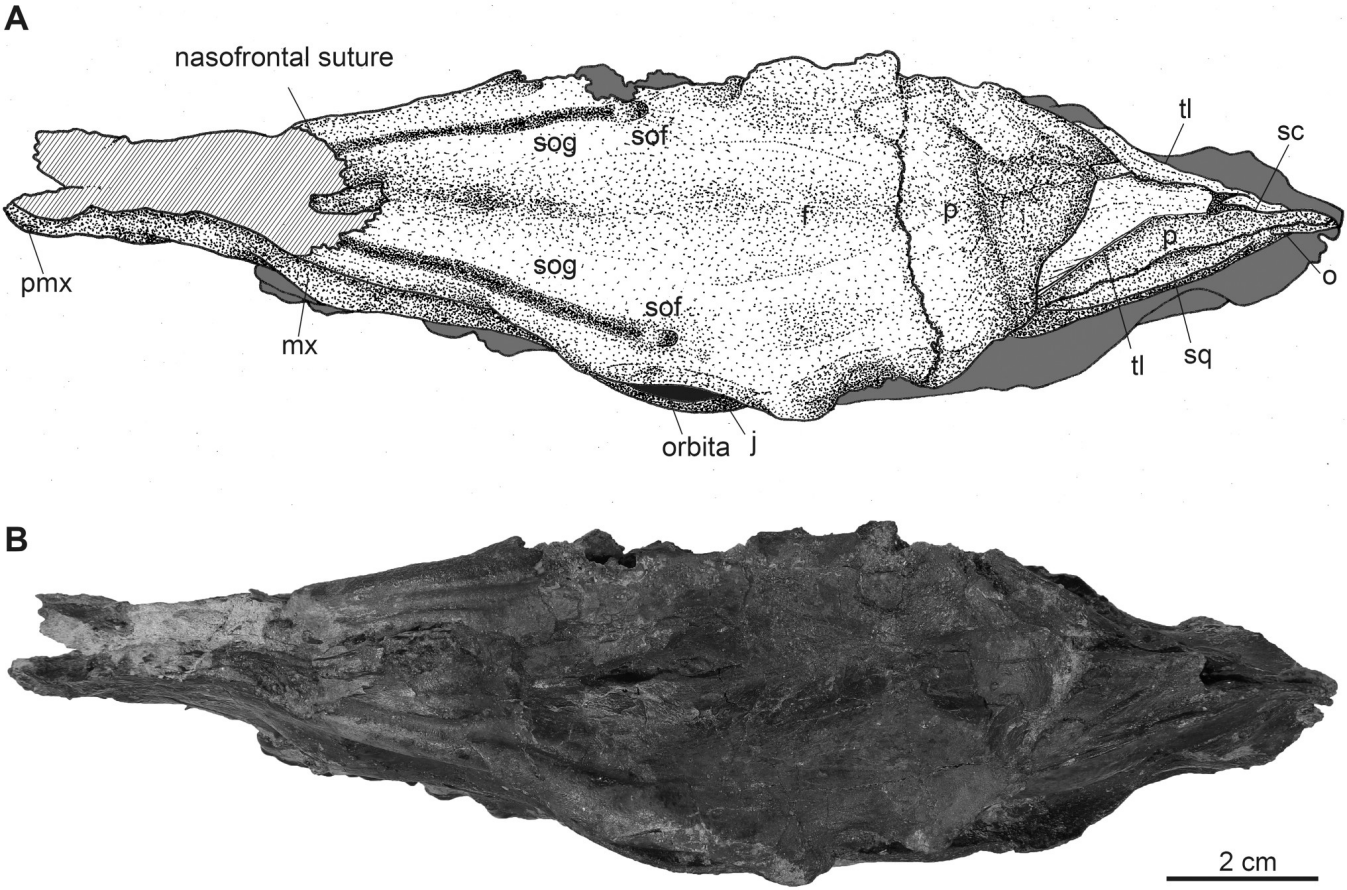
Complete female skull of *Dorcatherium naui* (GPIT/MA/18000-01) in dorsal view from the Hammerschmiede locality, Germany (HAM 4). (A) drawing, (B) photograph. Abbreviations: f, frontal; j, jugal; mx, maxillary; o, occipital; p, parietal; pmx, premaxillary; sc, sagittal crest; sof, supraorbital foramen; sog, supraorbital groove; sq, squamosal; tl, temporal lines.

**Fig 5:**
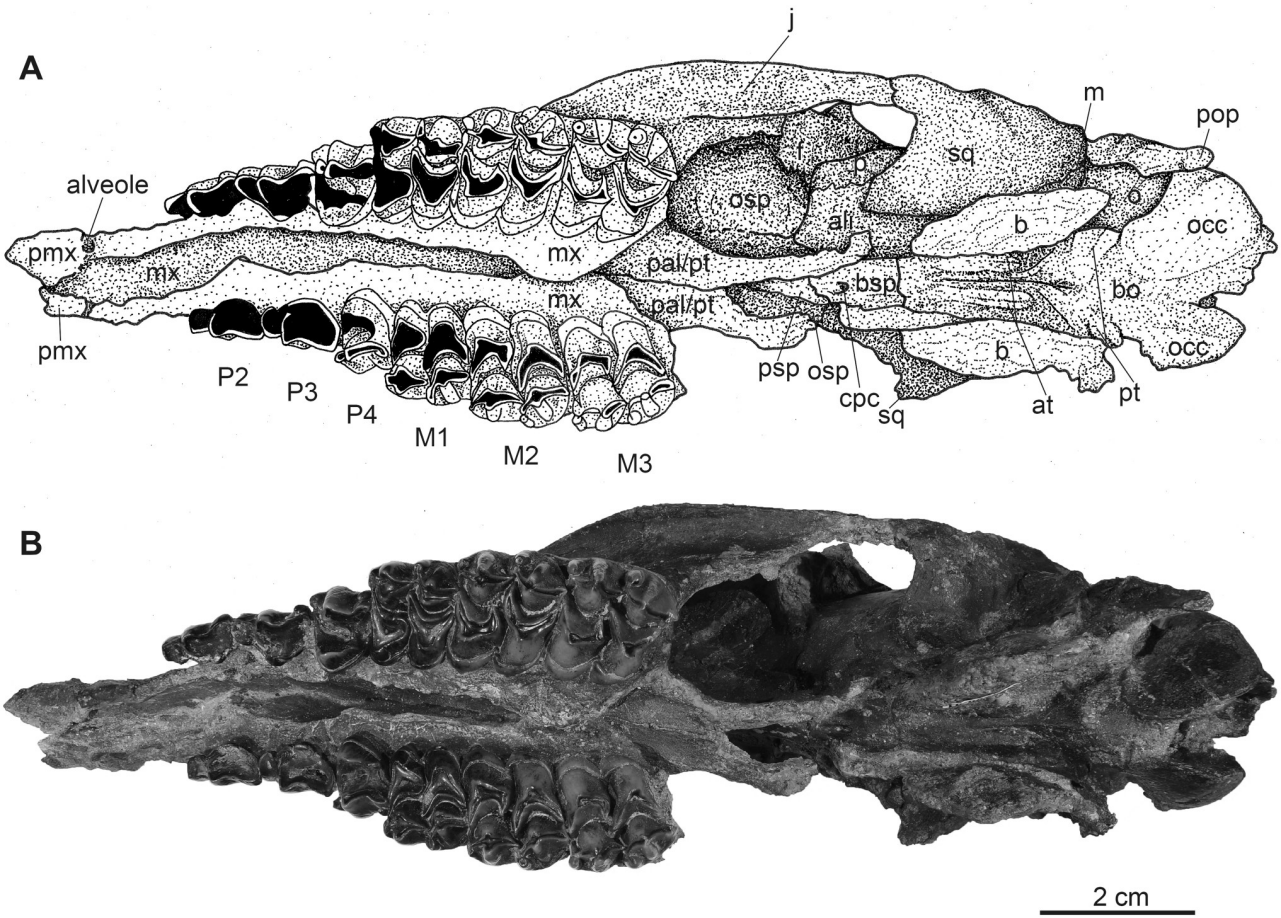
Complete female skull of *Dorcatherium naui* (GPIT/MA/18000-01) in ventral view from the Hammerschmiede locality, Germany (HAM 4). (A) drawing, (B) photograph. Abbreviations: ali, alisphenoid; at, anterior tuberosities; b, bulla tympanica; bsp, basisphenoid; bo, basioccipital; cpc, craniopharyngeal canal; f, frontal; m, mastoid process; mx, maxillary; o, occipital; occ, occipital condyle; osp, orbitosphenoid; p, parietal; pmx, premaxillary; pop, paroccipital process; psp, presphenoid; pt, posterior tuberosities; pt/pal, pterygoid/palatine; sq, squamosal.

**Fig 6:**
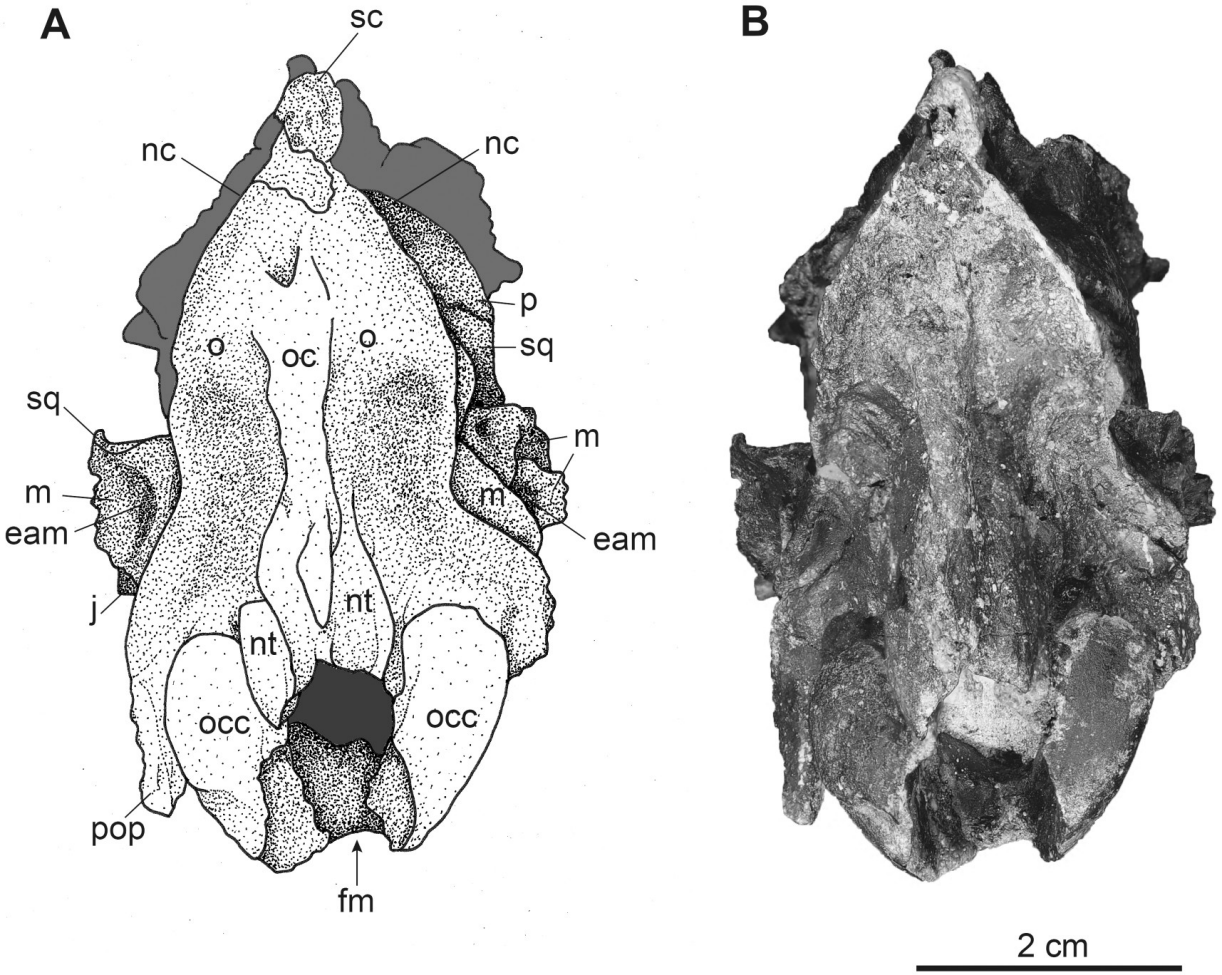
Complete female skull of *Dorcatherium naui* (GPIT/MA/18000-01) in posterior view from the Hammerschmiede locality, Germany (HAM 4). (A) drawing, (B) photograph. Abbreviations: eam, external auditory meatus; fm, foramen magnum; j, jugal; m, mastoid process; nc, nuchal crests; nt, nuchal tubercle; o, occipital; oc, occipital crest; occ, occipital condyle; p, parietal; pop, paroccipital process; sc, sagittal crest; sq, squamosal.

**Fig 7:**
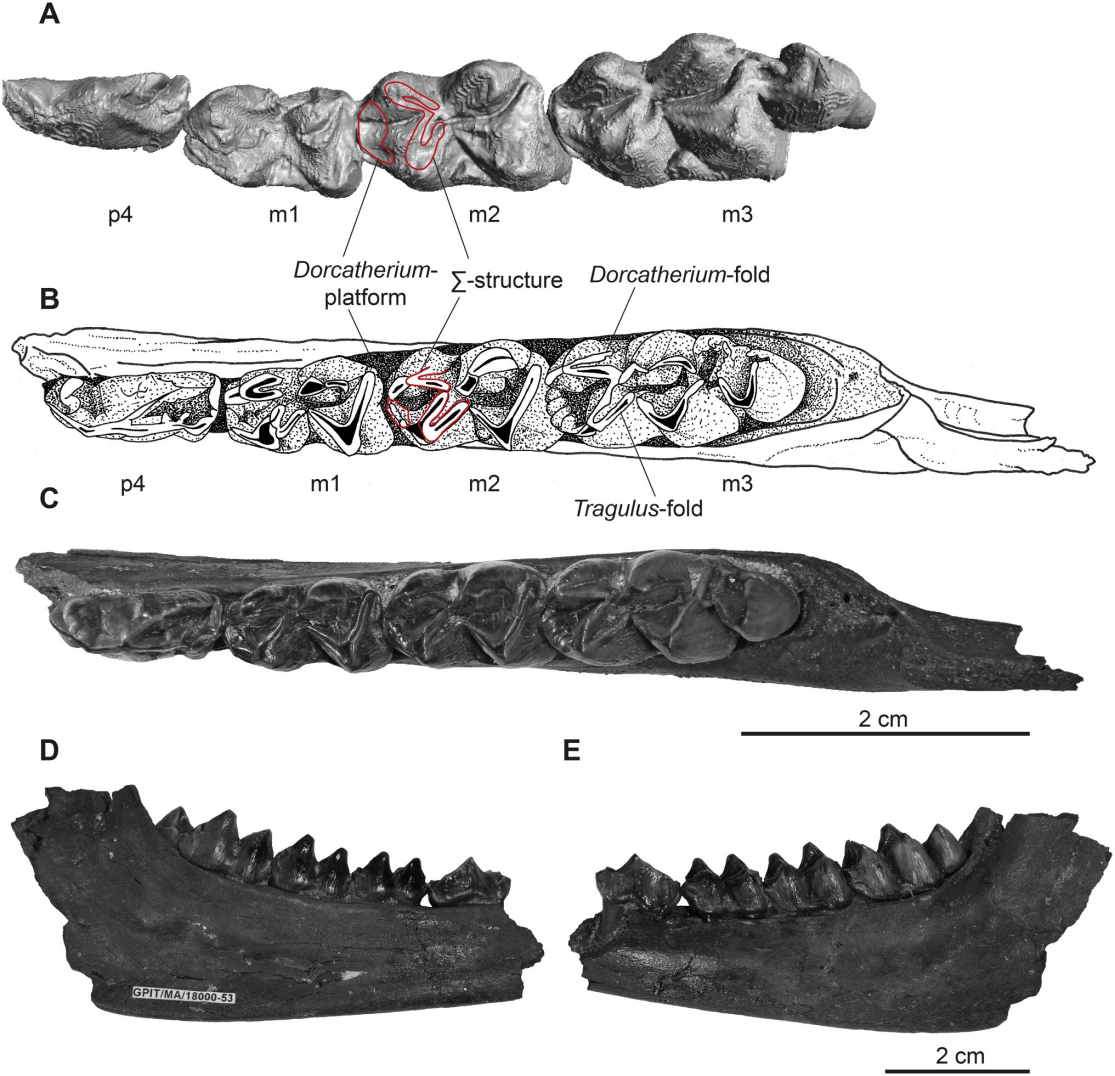
Lower dentition of *Dorcatherium naui*. The mandible GPIT/MA/18000-53 is associated to the skull GPIT/MA/18000-01. (A) for comparison: virtually isolated, left tooth row of NHMUK PV OR 40632 in occlusal view, (B) drawing of GPIT/MA/18000-53 in occlusal view, (C) GPIT/MA/18000-53 in occlusal view, (D) GPIT/MA/18000-53 in lingual view, (E) GPIT/MA/18000-53 in buccal view.

**Fig 8:**
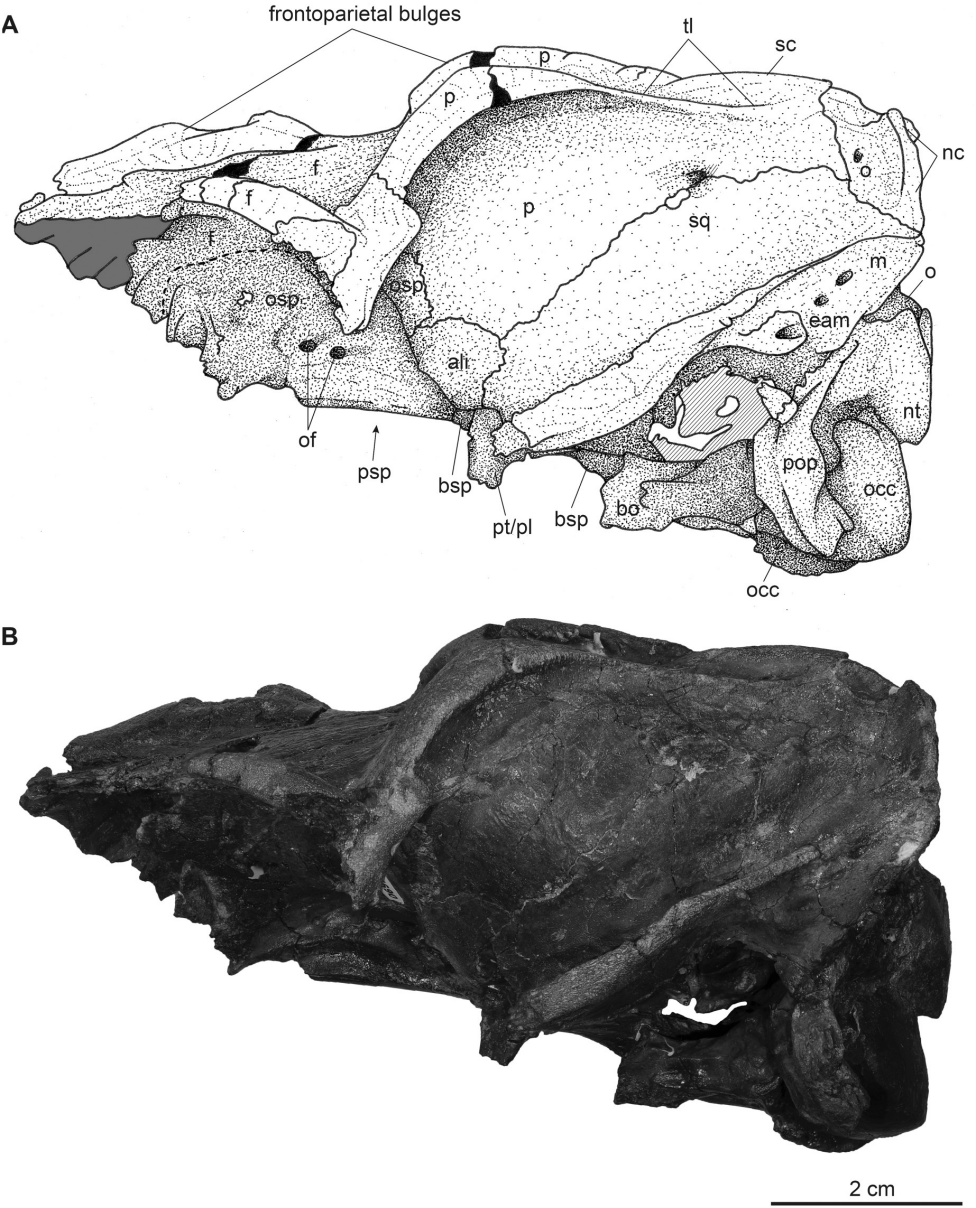
Partial male skull of *Dorcatherium naui* (GPIT/MA/17690) in lateral view from the Hammerschmiede locality, Germany (HAM 4). (A) drawing, (B) photograph. Abbreviations: ali, alisphenoid; bo, basioccipital; bsp, basisphenoid; eam, external auditory meatus; f, frontal; m, mastoid process; nc, nuchal crests; nt, nuchal tubercle; o, occipital; of, optic foramen; occ, occipital condyle; osp, orbitosphenoid; p, parietal; pop, paroccipital process; psp, parasphenoid; pt/pal, pterygoid/palatine; sc, sagittal crest; sq, squamosal; tl, temporal line.

**Fig 9:**
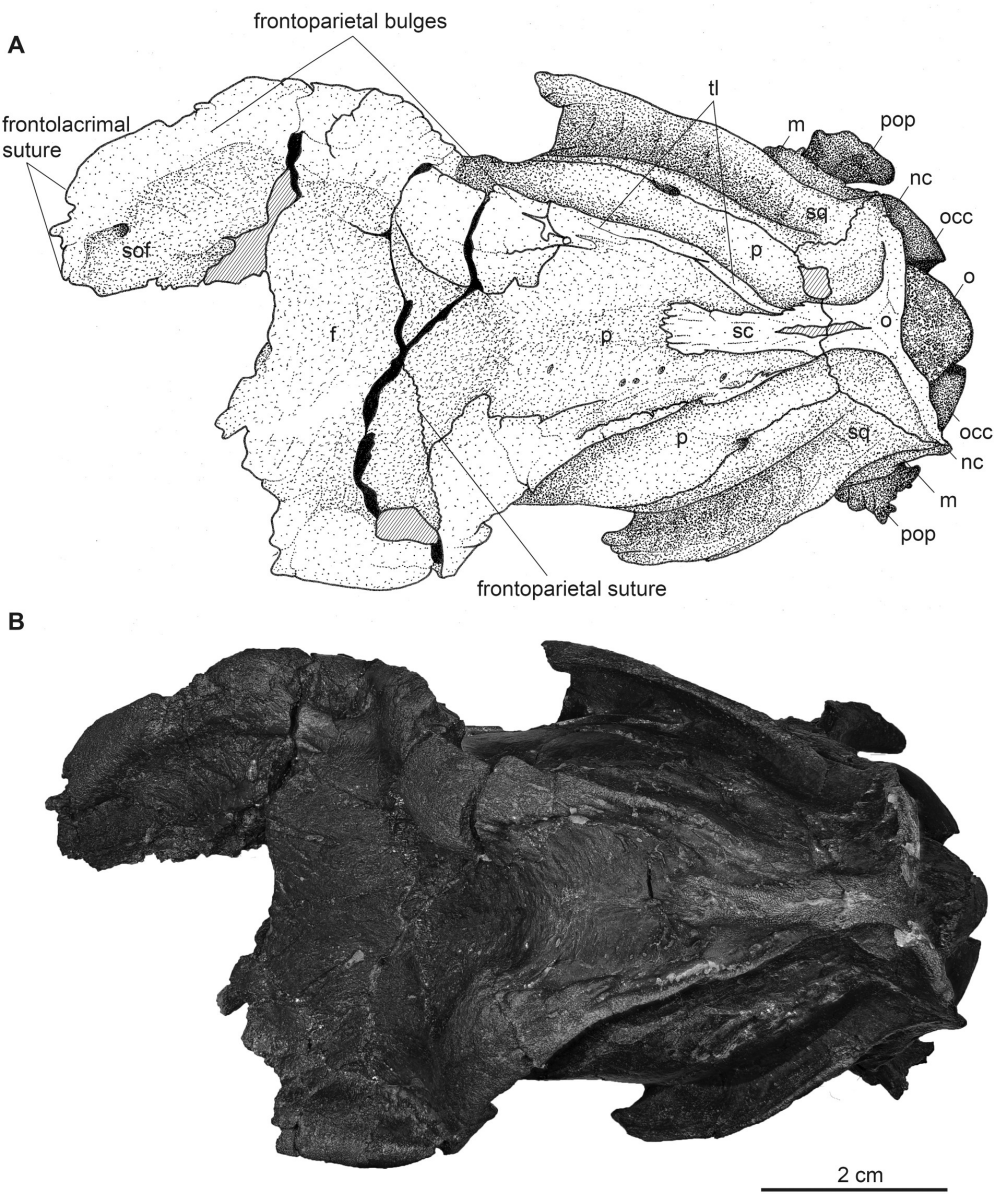
Partial male skull of *Dorcatherium naui* (GPIT/MA/17690) in dorsal view from the Hammerschmiede locality, Germany (HAM 4). (A) drawing, (B) photograph. Abbreviations: f, frontal; m, mastoid process; nc, nuchal crests; o, occipital; occ, occipital condyle; p, parietal; pop, paroccipital process; sc, sagittal crest; sof, supraorbital foramen; sq, squamosal; tl, temporal line.

**Fig 10:**
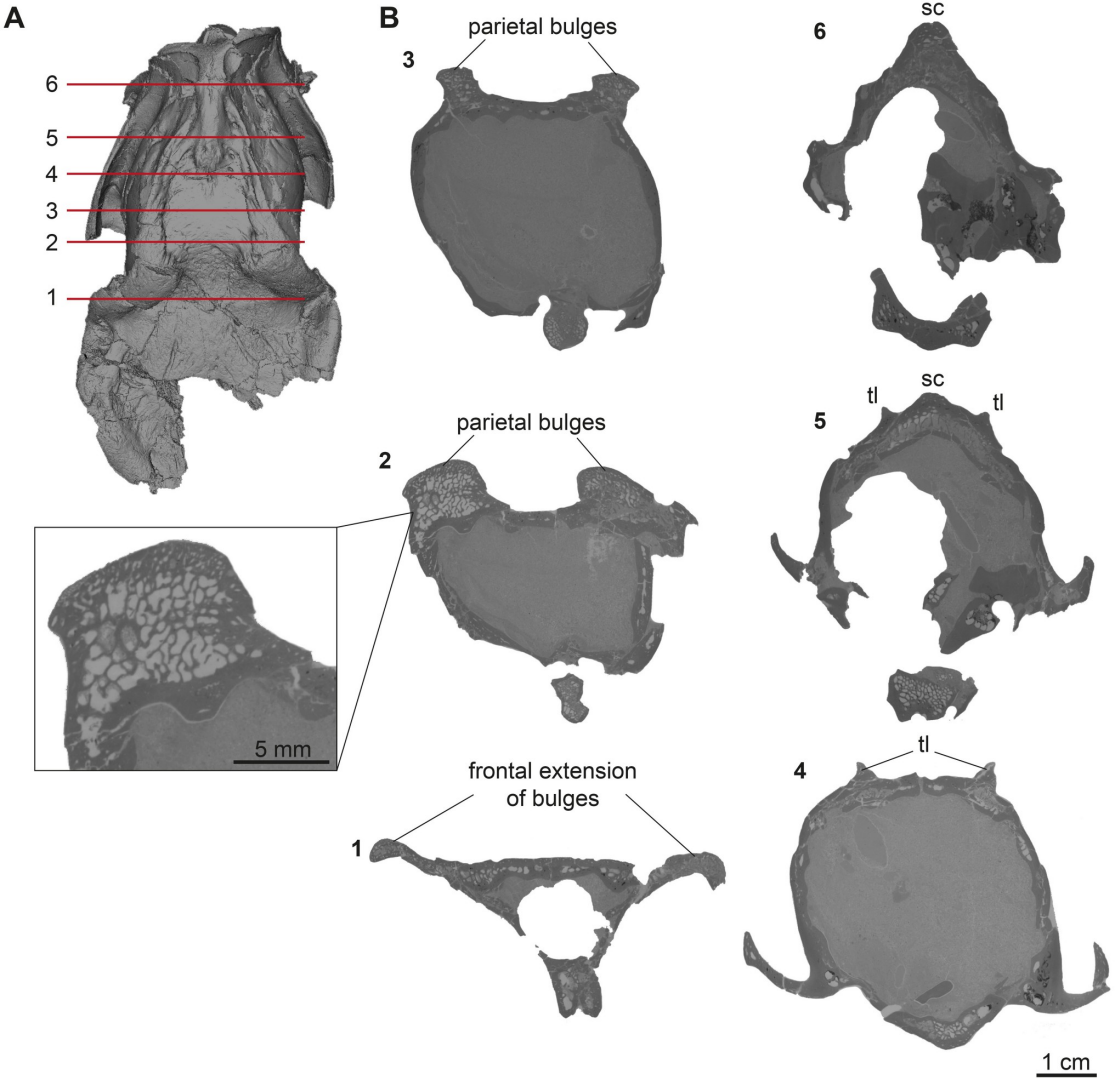
Cranial ornamentation of male *D*. *naui*. (A) Position of virtual cross section. (B) median plane μCT-slices of GPIT/MA/17690.

**Fig 11:**
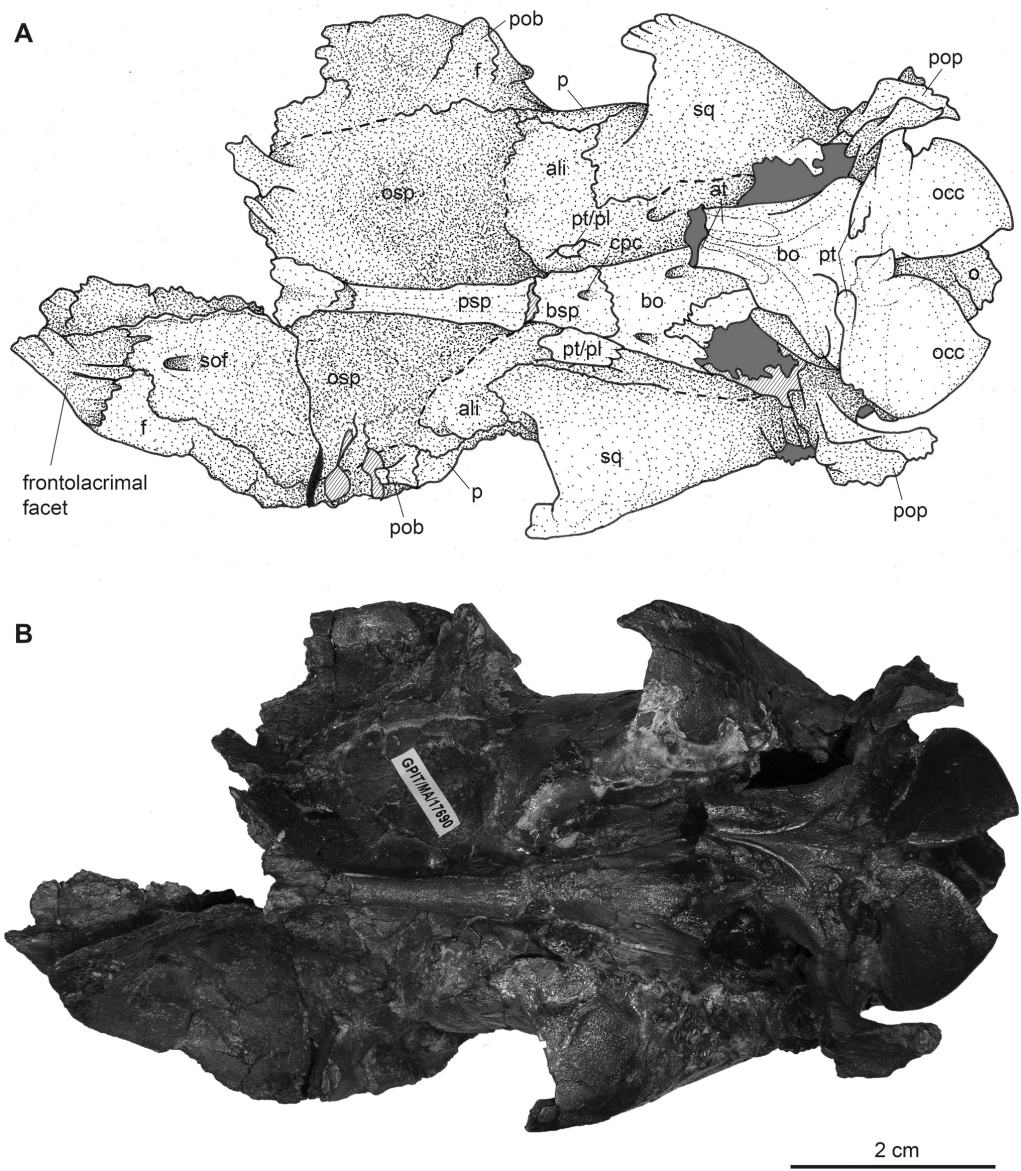
Partial male skull of *Dorcatherium naui* (GPIT/MA/ 17690) in ventral view from the Hammerschmiede locality, Germany (HAM 4). (A) drawing, (B) photograph. Abbreviations: ali, alisphenoid; at, anterior tuberosities; bsp, basisphenoid; bo, basioccipital; cpc, craniopharyngeal canal; f, frontal; o, occipital; occ, occipital condyle; osp, orbitosphenoid; p, parietal; pob, postorbital bar; pop, paroccipital process; psp, presphenoid; pt, posterior tuberosities; pt/pal, pterygoid/palatine; sof, supraorbital foramen; sq, squamosal.

**Fig 12:**
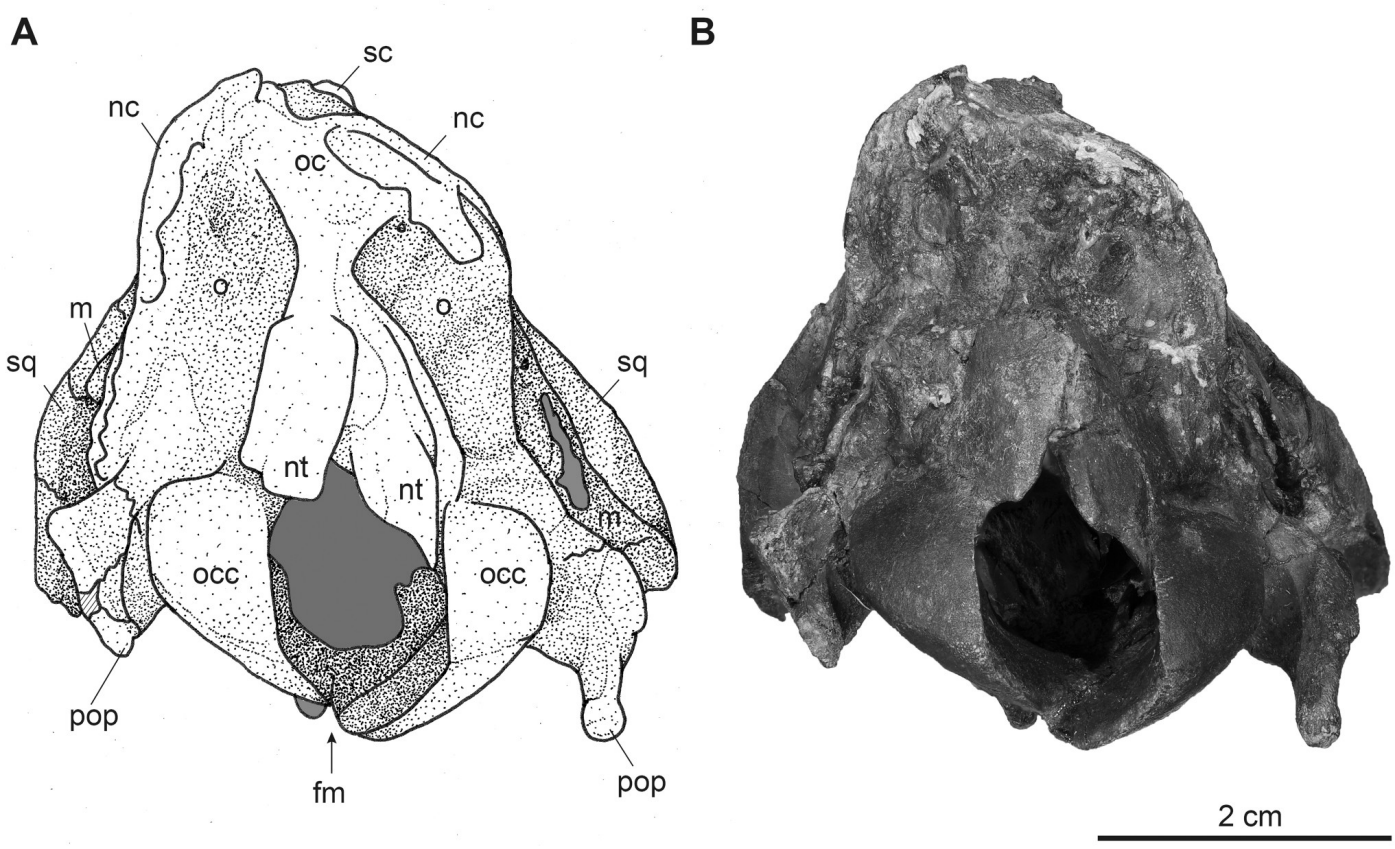
Partial male skull of *Dorcatherium naui* (GPIT/MA/17690) in posterior view from the Hammerschmiede locality, Germany (HAM 4). (A) drawing, (B) photograph. Abbreviations: fm, foramen magnum; m, mastoid process; nc, nuchal crests; nt, nuchal tubercle; o, occipital; oc, occipital crest; occ, occipital condyle; pop, paroccipital process; sc, sagittal crest; sq, squamosal.

### Remarks

The holotype of *Dorcatherium naui* was first described by Kaup [36] in a letter to Bronn. Kaup [36] described the specimen as different from other ruminants, because the mandible contains seven teeth instead of six. Furthermore, he described the teeth as similar to a deer. The holotype was later mentioned by Kaup and Scholl [37] and was finally displayed by Kaup (pl. 23, Fig 1-1b in [14]). After that, the holotype was lost, but one cast is stored in the NHMUK under the collection number NHMUK PV M 3714 and another one is stored in the SNSB-BSPG under the collection number SNSB-BSPG 1961 XIX 37. In the same paper, where the holotype was figured in 1839, Kaup (pl. 23c, Figs 1 and 2 in [14]) also displayed a complete male skull of *D*. *naui* (NHMUK PV OR 40632) with attached mandibles and atlas from Eppelsheim, Germany, but this specimen was described later in [13]. Now this skull is stored in the NHMUK and it represents the only other *Dorcatherium* skull from the type locality of *D*. *naui*.

## Skull morphology of *Dorcatherium naui*

NHMUK PV OR 40632 is a complete skull with two attached mandibles and two large upper canines (Figs 1 and 2). The skull is laterally compressed with a heavy diagenetic depression of the left preorbital part. This depression is visible in the μCT scan, the cast housed in the GPIT, and the original drawing of Kaup (pl. 23A, Figs 1 and 2 in [14]). GPIT/MA/17690 and GPIT/MA/18000-01 are both from the Hammerschmiede locality. In GPIT/MA/17690 the preserved parts of the skull are, besides some minor diagenetic anteroposterior and dorsoventral distortion, well-preserved. The preorbital portion of the skull is not preserved (Figs 8 and 9) and therefore the premaxillary, maxillary, nasal, lacrimal, and jugal are not described. In GPIT/MA/18000-01, the left side of the skull is well-preserved, except for some diagenetic alteration (Fig 3), whereas the right side is laterally crushed and the paroccipital process and zygomatic arch with most of the jugal are missing. The premaxillary is partially preserved, while the nasals are missing completely (Fig 4). Furthermore, the teeth P2–M3 of the left and right maxillaries are present. A small alveole for the reduced canine is visible at the anterior most part of the left maxillary (Fig 5).

In general, all skulls are anteroposteriorly elongated and the snout is tapered; the brain cavity is low and prolate. GPIT/MA/18000-01 has a postfrontal length of 53.8 mm and a condylobasal length of 157.1 mm and GPIT/MA/17690 possesses a slightly larger postfrontal region that is 56.8 mm in length (Table 1). In comparison the skull from Eppelsheim has a postfrontal length of ~65 mm and a condylobasal length of ~149 mm.

**Table 1:** Skull measurements of *Dorcatherium*.

Specimen number	Species and locality	a	b	c	d	e	f	g	h	i	j	k	l	m	n	o	p	q
**NHMUK PV OR 40632, cast GPIT/MA/03653**	*D*. *naui* Eppelsheim	~149	/	/	/	/	/	/	~33	~43	~91	~37	~56	~65	21.1	~65	/	9.2
**GPIT/MA/18000-01**	*D*. *naui* Hammerschmiede (HAM 4)	157.1	~47	29.8	30.6	~28	~35	~11	~30	~36	~92	~48	~63	72.5	21.4	53.8	14/~4	1.9
**GPIT/MA/17690**	*D*. *naui* Hammerschmiede (HAM 4)	/	53.8	/	/	33.2	45.4	~12	38.5	/	/	36.7	51.9	/	/	56.8	26/3	/
**NMA-Walda 2**	*D*. *crassum* Walda 2	173	80	38.3	36.4	35	44.6	17.9	46.6	52	102.4	37.9	51.9	72.5	21.2	43	29/4.2	/
**NMA-2012-1/2131 (Thierhaupten)**	*D*. *crassum* Thierhaupten	153	84.3	27.5	34.1	29.9	38.4	15.2	50	46.9	/	34	45	66.3	/	/	/	/

All measurements are in mm. Parameters and measurements of *D*. *crassum* (Walda 2 and Thierhaupten) after [11] except (o), (p) and (q); (a) Condylobasal length; (b) Zygomatic width; (c) Largest dorsoventral diameter of orbits; (d) Largest anteroposterior diameter of orbits; (e) Occipital condylar width; (f) Paroccipital width; (g) Latero-lateral diameter of foramen magnum; (h) Width of braincase; (i) Interorbital width; (j) Maxillar lateral length; (k) Height dorsal ophistion; (l) Height dorsal basion; (m) Length of upper tooth row; (n) Length of upper diastema; (o) postfrontal length; (p) length/height of sagittal crest; (q) diameter of upper canine alveole. Numbers including tilde represent approximations due to the preservation of the specimen.

### Premaxillary

The premaxillary is well preserved in the skull from Eppelsheim (Figs 1 and 2) and partially preserved in GPIT/MA/18000-01 (Fig 3). It forms the anterior part of the snout and continues beneath the anterior extension of the nasal in the skull from Eppelsheim.

### Maxillary

In GPIT/MA/17690, the maxillaries are not preserved and in the skull from Eppelsheim these bones are heavily distorted due to lateral deformation. In contrast, in GPIT/MA/18000-01, both maxillaries are exposed, but the left one is better preserved (Fig 3). In lateral view, the maxillary is the largest bone of the skull reaching far posteriorly to the level of the posterior part of the closed orbital margin (Fig 3). Anteriorly, where it is sutured to the remains of the premaxillary, it is slightly concave in GPIT/MA/18000-01. Dorsally, the sutural facet for the missing nasal is exposed and posterodorsally, the maxillary is in contact with the frontal, lacrimal and jugal. Above the P2, the infraorbital foramen is visible in GPIT/MA/18000-01 and NHMUK PV OR 40632. In ventral view (Fig 5), the maxillary forms by far the largest part of the snout. Here, the maxillary is crushed in GPIT/MA/18000-01, similar to the specimen from Eppelsheim. It is sutured anteriorly with the premaxillary and posteriorly with the palatine. The anterior part of the ventral maxillary contains the alveolus for the canine. This alveolus is very small in GPIT/MA/18000-01 by being only 1.9 mm in diameter compared to the skull from Eppelsheim, where it is 9.2 mm in diameter (Table 1).

### Nasal

The skull from Eppelsheim is the only one that preserves the nasal (Figs 1 and 2). In lateral view, the nasal is in contact with the posterodorsal margin of the premaxillary and extends to the anterior end of the latter as already noticed by Rössner [15]. The nasofrontal suture is hardly visible, but seems to be W-shaped with the sagittal process facing anteriorly.

### Lacrimal

In lateral view, in the skull from Eppelsheim and GPIT/MA/18000-01 (Figs 1–3), the lacrimal forms the anterior rim of the orbital margin and is anteroposteriorly elongated. It is sutured dorsally with the frontal and inside the orbita with the orbitosphenoid. Ventrally it contacts the maxillary and jugal. A single lacrimal foramen is visible inside the orbita. A lacrimal fossa is absent.

### Skull roof (frontal and parietal)

The fused frontals form a major part of the skull roof in all three specimens (Figs 2, 4 and 9). In dorsal view, they are confined anteriorly by the anterior lacrimomaxillary suture and posteriorly by the frontoparietal suture. Anteriorly, the suture is W-shaped with a prominent anteriorly pointed sagittal prolongation. At the lateral part of the anterior skull roof, the frontals contain the supraorbital foramina originating inside the orbita. Both foramina are situated at the level of the anterior part of the orbita next to the orbital margin (Figs 2, 4 and 9). They continue as parallel supraorbital grooves and run anteriorly towards the nasofrontal suture in the skull from Eppelsheim and the GPIT/MA/18000-01.

While the frontal constitutes the anterior part of the skull roof, the parietal forms the posterior part and is connected posteriorly to the occipital (Fig 4). In GPIT/MA/17690, in dorsal view, the anterior part of the frontals expands laterally, forming a slightly concave plateau encompassed by flat and broad bony bulges above the orbita. These bulges become more prominent, robust, and set off with a rough surface texture at the parietals. Here, they bend medially to constrict the domed anterior parietal, forming the plateau until they merge with the crest-like temporal lines (Figs 8 and 9). Due to the lateral distortion of the skull from Eppelsheim, these structures are poorly preserved. They are deformed on the left side and are broken off at the frontal on the right side (Figs 1 and 2).

Posteriorly, the temporal lines of GPIT/MA/17690 enclose the parietal plateau until they run asymptotic to the well-developed, flat, and broad sagittal crest to merge with the posterior part of the latter. In the skull from Eppelsheim, these structures are well-visible despite the deformation of the skull roof. Fig 2 shows that especially the right side of the skull exposes massive parietal bulges that are set off from the skull roof. In Fig 2C the posterior view of the skull shows two prominent parietal bulges confining towards the center of the skull roof, forming an overhang above the lateral parietal. The internal structure of the bulges is very porous surrounded by compact bone. This is well visible in Fig 10B (cross section 2) at the thickened part above the frontal.

The sagittal crest is best visible in GPIT/MA/17690. Here, its anterior half proceeds onto the well-developed and prominent parietal plateau. The sagittal crest is 26 mm long and 3 mm high. In the specimen from Eppelsheim a sagittal crest is visible, but due to the poor preservation its morphology cannot be accurately described.

In contrast, GPIT/MA/18000-01 exposes no such frontoparietal bulges. Instead, the parietal is enclosed by the crest-like temporal lines surrounding a small plateau at the posterior part of the bone. The temporal lines merge posteriorly into a well-developed sagittal crest. The sagittal crest is 14 mm long and 4 mm high at its maximum. The temporal lines form the dorsal margin of the fossa for the temporal muscle.

### Squamosal

The parietal and squamosal form the lateral wall of the braincase, with the squamosal forming the ventral part of the lateral wall of the braincase in all three specimens (Figs 2, 3 and 8). Anteriorly, the squamosal is sutured with the alisphenoid, dorsally with the parietal, posteriorly with the occipital, and ventrally with the mastoid process. At the central level of the squamosoparietal suture, a foramen is situated. The squamosal expands anterolaterally to contact the jugal at the level of the alisphenoid and together they form the zygomatic arch. The ventrolateral squamosal extension is concave, anteroposteriorly elongated, and furthermore forms the ventral margin of the fossa for the temporal muscle. This ventrolateral squamosal process shows a deep insertion area, formed like a pit in GPIT/MA/18000-01. In GPIT/MA/17690, a small depression is visible instead, while this structure is not visible in the skull from Eppelsheim (Fig 9) due to preservation.

### Nuchal crests

Posteriorly, the squamosal forms a part of the nuchal crests (Figs 2–4, 8 and 9). The latter originate from the sagittal crest, diverge ventrally and represent attachment zones for the neck musculature. Furthermore, the nuchal crests are formed by the occipital and mastoid processes and follow the course of the occipital suture until they merge into the paroccipital process. They are more prominent in the GPIT/MA/17690 than in GPIT/MA/18000-01.

### Alisphenoid

Anterior to the squamosal, the alisphenoid is situated posteriorly to the orbita and medially to the zygomatic arch (Figs 3 and 8), which is well-visible in both skulls from Hammerschmiede. It forms a part of the lateral wall of the braincase. The alisphenoid is sutured anteriorly with the orbitosphenoid and posteriorly with the parietal and squamosal. In ventral view, the alisphenoid continues beneath the tympanic bulla and is confined by the basioccipital and basisphenoid (Figs 5 and 11).

### Jugal

The jugal is preserved in GPIT/MA/18000-01 and the skull from Eppelsheim (Figs 1–3). It is an elongated, slender, tripartite bone. The anterior process is sutured with the maxillary and lacrimal and posteriorly, it forms the flattened ventral margin of the orbita. The dorsal process is sutured to the frontal and together they form the postorbital margin. The posterior process of the jugal is sutured with the squamosal and forms the posterior part of the zygomatic arch. The jugal is characterized by a prominent horizontal edge for the attachment of musculature.

### Orbita

The lateral facing orbitae are situated dorsal to the jugal in GPIT/MA/18000-01 and the skull from Eppelsheim. In the latter the left orbita is small and oval if compared to the right one due to compression of the skull. In GPIT/MA/17690 the ventral margin of the orbitae is missing, but the dorsal margins are exposed.

In contrast, the left orbita of GPIT/MA/18000-01 is well-preserved and not compressed. It is large compared to the rest of the skull (Fig 3). The outline of the orbita is round to slightly rectangular. Anteriorly, the orbital margin is formed by the lacrimal, dorsally by the frontal, posteriorly by the postorbital bar, consisting of frontal and jugal, and ventrally by the anteroposteriorly elongated part of the jugal.

Internally, the orbita is formed by the lacrimal, frontal, and orbitosphenoid. In general, inside the orbita, four foramina are visible: the infraorbital foramen, the supraorbital foramen, the optic foramen, and the foramen orbitorotundum. At the anteriormost part, which is not preserved in GPIT/MA/17690, but instead in NHMUK PV OR 40632, the infraorbital foramen is situated along the frontolacrimal suture and connects the internal orbita with the nasal capsule. It contains the maxillary nerve, a branch of nerve (VII) that enters the nasal cavity inside the anterior part of the orbita and exits the nasal cavity at the maxillary. The anterodorsal part of the internal orbita contains the supraorbital foramen pinching through the internal wall of the orbita. It is the connection between the supraorbital foramen at the dorsal skull roof and the eye capsule and contains the ophthalmic branch of the trigeminal nerve (VI) together with the frontal artery and frontal vein.

Inside the posterior orbita the optic foramen is situated at the orbitosphenoid. The optic foramen contains the optic nerve (II). The largest foramen inside the orbita is the foramen orbitorotundum, situated posterior to the optic foramen and ventrally to the alisphenoid, inside the orbitosphenoid. It is a special characteristic of ruminants and the result of fusion of the foramen rotundum and the fissura orbitalis [38]. The foramen contains the ophthalmic nerve (VI), the nerves for the eye musculature consisting of the oculomotor nerve (III), trochlear nerve (IV), and abducent nerve (VI), and the maxillary nerve (VII) [39]. The foramen orbitorotundum connects the brain case with the internal orbita.

### Tympanic region

The tympanic region comprises the posterodorsally extending mastoid process. The auditory bullae are poorly preserved in all specimens and are even broken off in GPIT/MA/17690 (Figs 3 and 5). The mastoid is dorsally confined by the squamosal and posteriorly by the occipital. Mastoid and occipital are separated by the nuchal crests and the paroccipital process. Dorsally to the tympanic bullae, the foramen for the external auditory meatus is well-visible on both sides of GPIT/MA/18000-01, as well as GPIT/MA/17690. Posterodorsally to the foramen for the external auditory meatus, two smaller foramina are situated (Figs 3 and 8).

### Occipital

In the specimen from Eppelsheim, the occipital is heavily distorted and compressed, so that the foramen magnum is barely visible (Fig 2). In both specimens from Hammerschmiede the occipital is well-preserved and enclosed laterally by the nuchal crests. Dorsally, parts of the occipital extend anteriorly to the nuchal crests onto the lateral side of the skull. This area is larger in GPIT/MA/17690 than in GPIT/MA/18000-01. In posterior view, the occipital is slightly triangular and shows two rugose attachment surfaces for the neck musculature dorsally to the occipital condyles. Although the occipital is laterally compressed, the straight occipital crest is visible in all three specimens of *D*. *naui* (Figs 2, 6 and 12). The keel proceeds dorsoventrally and merges into two well-developed nuchal tubercles. The occipital condyles are dorsally set off from the occipital ventrally to the well-developed nuchal tubercles. Laterally to the occipital condyles, the elongated, slender paroccipital processes are visible.

### Basicranium

In ventral view, the basicranium is well-preserved and comprises the basioccipital and the basisphenoid preserved in all three specimens (Figs 1, 5 and 11). The basioccipital is slender, V-shaped, and laterally confined by the alisphenoid and the tympanic bullae. Anteriorly, the suture between the basioccipital and the basisphenoid is straight. Posteriorly, the occipital condyles merge with the occipital. Anterior to the occipital condyles, two weakly-developed posterior tuberosities are visible that are slightly elevated and form the laterally oriented crests. Slightly anterior, the paired anterior tuberosities form elongated ridges proceeding anteroposteriorly. They have a steep posterior and a flattened anterior part. At the center of the anterior tuberosities, a smooth keel is exposed that vanishes halfway towards the basisphenoid.

The basisphenoid is elongated, rectangular, and extends the basioccipital anteriorly but its size is relatively small compared to the basioccipital. The basisphenoid is bordered laterally by the alisphenoid and the palatine/pterygoid. The basisphenoid contains a prominent foramen for the craniopharyngeal canal in its center [40]. Anterior to the basisphenoid, the presphenoid is slender and anteroposteriorly elongated. The anterior part of the presphenoid becomes thin, narrow, and continues beneath the secondary palate.

### Palatine

In ventral view, the palatine is well-visible in GPIT/MA/18000-01. It forms a triangular plateau posterior to the maxillary (Fig 5). The lateral parts are crushed and a suture to the pterygoid is not visible. The palatine/pterygoid forms the lateral wall of the choana and connects with the alisphenoid posteriorly.

### Maxillary dentition

GPIT/MA/18000-01 as well as the skull from Eppelsheim preserve the teeth P2–M3 on both sides. In GPIT/MA/18000-01, a small alveolus (1.9 mm in diameter) for the rudimentary upper canine is visible (Figs 3 and 5). In contrast, the skull from Eppelsheim shows large saber-like upper canines with an alveolus size of 9.2 mm (Figs 1 and 2). here, the alveoli extend posteriorly up to the level of the nasomaxillary suture and the anterior margin of the P3. The cross-section of the tooth is drop-shaped.

P2–M3 are bunoselenodont with a well-developed buccal relief. The buccal wall has wrinkled enamel, with straight and parallel folds. The central fold is large compared to the smaller, lateral ones. The lingual wall of P4–M3 is marked by a continuous cingulum reaching from the mesial protocone to the distal metaconule in the molars. The cingulum of P4 surrounds the lingual cone mesiodistally.

In occlusal view, the premolars, P2 and P3 are quite similar to each other but differ from P4 in morphology, as P2 and P3 are longer than wide with well-developed buccal cones. The P4 is wider than long and exposes a deep fossa and a lingual cingulum. The P2 is slightly more elongated than the P3. The P4 contains a broad fossa, its central fold is well-developed, and it connects to the posterobuccal cone. In comparison to P2 and P3, the P4 shows a well-developed lingual cone, encompassing the fossa lingually.

The upper molars are square-shaped and increase in size from M1–M3 (Figs 1 and 5). They contain well-developed styles. The metaconule is V-shaped and the premetaconule crista is longer than the postprotocrista, which reaches the posterolingual wall of the paracone. The postprotocrista is not connected to the premetaconule crista, because the distal postprotocrista folds mesiobuccally. All molars show prominent para—and mesostyles but weak metastyles. The development of the metastyle increases from M1 to M3.

### Mandibular dentition

The skull from Eppelsheim preserves remains of the left and right mandible. Both, the mandible ramus, as well as the symphysis are badly preserved (Fig 1). They expose remains of the incisors and the p1–m3. GPIT/MA/18000-53 preserves only the central part of the left mandible, containing the p4–m3 (Fig 7). The teeth p1–m3 are bunoselenodont with a weak lingual relief and wrinkled enamel. In occlusal view, the p1 is monocuspid. The p2 and p3 are generally mesiodistally longer than wide. The p2 is bicuspid with the mesolabial conid being larger than the posterolabial conid. The p3 has a central mesolabial conid, which is the largest cusp. The anterior conid forms a small anterior valley, which is slightly bent anterolingually. The p4 shows a central mesolabial conid, where the posterolingual and posterolabial conid originate. They continue straight and parallel towards the distal part of the p4.

The lower molars show a pair of crests posterior to the metaconid; the internal postmetacristid and well-developed external postmetacristid (*Dorcatherium*-fold, *sensu* [30]). The internal postmetacristid connects to the internal postprotocristid. The internal postprotocristid merges into the external postprotocristid (*Tragulus*-fold, *sensu* [34]) buccally. Together, the postmetacristids and postprotocristids form a Ʃ-structure. Anterior to the Ʃ-structure, the well-developed *Dorcatherium*-platform is visible, formed by the well-developed preprotocristid that turns lingually to contact a very small premetacristid.

Anteriorly, the preprotocristid and premetacristid encompass the anterior fossa. The mesostylid and metastylid are absent. The posterior fossa is surrounded by the preentocristid connecting to the internal postmetacristid and the postentocristid forming a slight lingually bent wall, as well as the prehypocristid and posthypocristid buccally. The posthypocristid and postentocristid are not connected, because the posterior postentocristid is bent buccally. The m3 contains a well-developed back fossa, formed by the hypoconulid. The entoconulid is weakly-developed and therefore the back fossa opens anterolingually.

### Taxonomical attribution

Both specimens from Hammerschmiede, as well as the skull from Eppelsheim can be assigned to the family Tragulidae based on the presence of a typical Ʃ-structure (*Tragulus*–and *Dorcatherium*-fold *sensu* [30, 34]) in the lower molars [2] and a bunoselenodont occlusal pattern (Fig 7), as well as to *Dorcatherium* based on the following combination of characters: (I) presence of p1 in NHMUK PV OR 40632, in combination with (II) the presence of the *Dorcatherium*-platform in the lower molars (*sensu* [35]), and (III) a complex posterior valley of the p4 (*sensu* [8]). Furthermore, GPIT/MA/18000-01 and the skull from Eppelsheim share the following features of the selenodont lineage that includes *D*. *guntianum*, *D*. *jourdani*, *D*. *puyhauberti*, *and D*. *naui*: high degree of selenodonty with reduced external postmetacristid [30, 34] in the lower molars and well-developed cristids, flat main cusps (*sensu* [34]), and prominent sickle-shaped cones and conids (*sensu* [7]). Therefore, these skulls are clearly distinguishable from *D*. *crassum*, *D*. *vindebonense*, and *D*. *penecki*.

**Fig 13:**
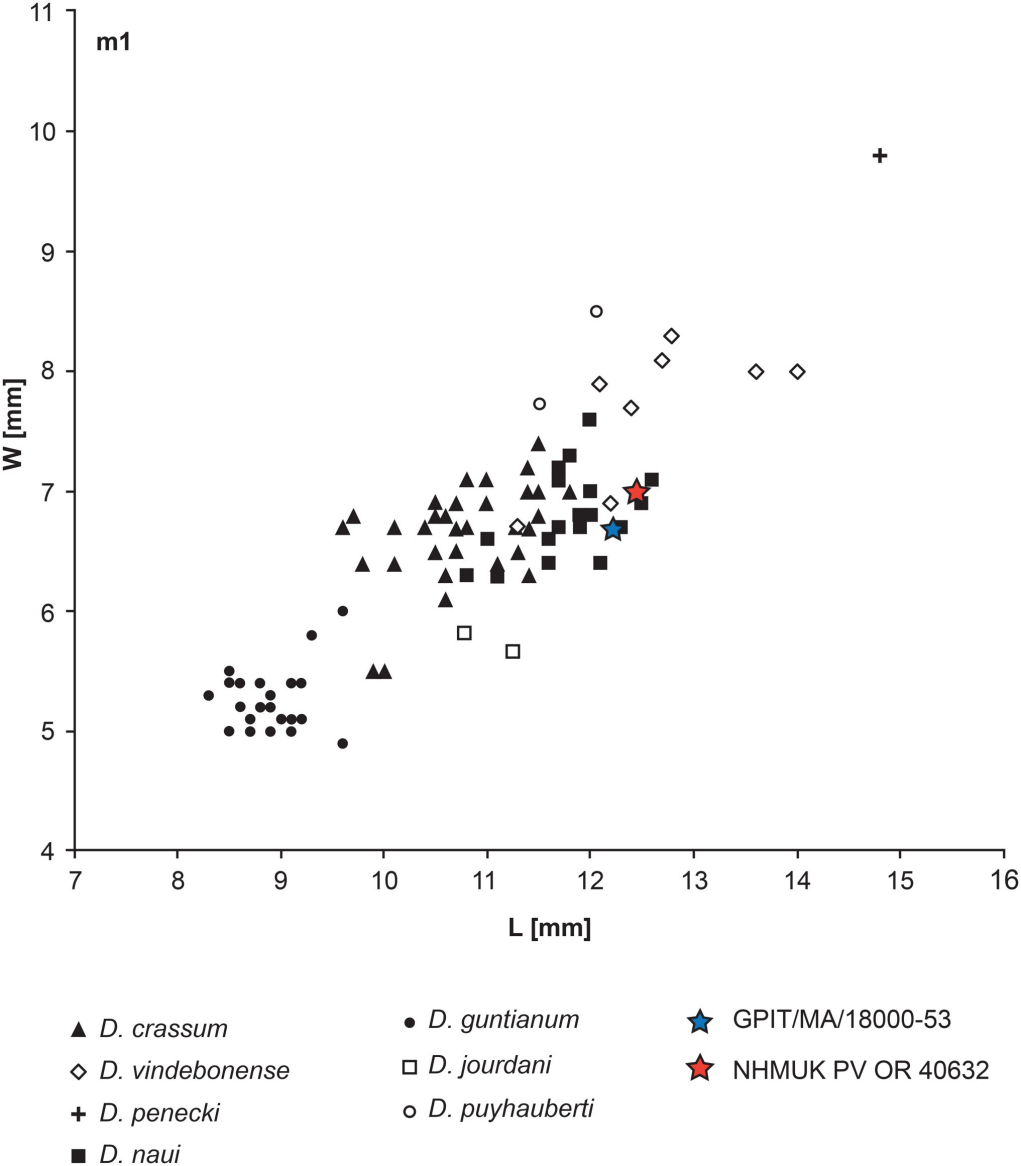
m1 tooth metrics of *Dorcatherium*. Data based on [31]. Position of GPIT/MA/18000-53 and NHMUK PV OR 4632 indicated with a colored star. W = maximum anterior width and L = maximum length.

Among the selenodont lineage *D*. *guntianum*, *D*. *jourdani*, *D*. *puyhauberti*, and *D*. *naui* differ in the size (length and width) of their teeth. The m1 of *D*. *guntianum* is clearly distinct from GPIT/MA/18000-53 and NHMUK PV OR 40632 by its much smaller length (Fig 13, maximum length of *D*. *guntianum* is about 9.8 mm and the minimum length of *D*. *naui* is about 11.0 mm). The width of the m1 of *D*. *jourdani* is smaller, whereas it is larger in *D*. *puyhauberti* if compared to GPIT/MA/18000-53 and NHMUK PV OR 40632. Hence, GPIT/MA/18000-53 and NHMUK PV OR 40632 are comparable in size with other known specimens of *D*. *naui* [8, 9, 31] and perfectly match the size range of this species. The combination of the metrics of the m1 (Fig 13), as well as the selenodont occlusal morphology of GPIT/MA/18000-53 and NHMUK PV OR 40632, support their assignment to *D*. *naui*.

Although GPIT/MA/17690 preserves no teeth, the specimen can still be assigned to Tragulidae based on the morphology of the posterior part of the skull and the morphology of the basicranium. In lateral view, the posterior part of the skull is low, prolate, and elongated posterodorsally, marked by well-developed nuchal, as well as sagittal crests typical for tragulids. Furthermore, a large temporal fossa compared to the rest of the skull is encompassed by the temporal lines and sagittal crest dorsally, the well-developed ventral process of the squamosal ventrally, as well as by the prominent nuchal crests posteriorly. This condition is not present in suids (*Sus scrofa*), cervids (*Capreolus capreolus*, *Muntiacus muntjak*), moschids (*Moschus* sp.), and bovids (*Bos taurus*, *Capra aegagrus*, and *Ovis gmelini*; all GPIT unnumbered). Morphologically similar conditions are visible in the moschid and suid specimens mentioned above; however, in suids the sagittal crest and temporal lines are weaker developed and in moschids, the braincase is more globular, rounded, and the posterior skull roof tapers posteriorly.

Furthermore, the morphology of the basicranium supports the allocation of GPIT/MA/17690 into Tragulidae, because of the presence of a large craniopharyngeal foramen, a slender basicranium with prominent, anteroposteriorly elongated anterior tubercles, and a smooth, central keel. This condition is visible in all three specimens described herein, as well as in the extant *Tragulus* (GPIT/MA/12997) and *D*. *crassum* from Thierhaupten [10].

## Comparison

### Distinguishing *D*. *naui* from *D*. *crassum*

In general, the only three available skulls of *D*. *naui* from Hammerschmiede and Eppelsheim, as well as the three known skulls of *D*. *crassum* from Thierhaupten, Walda 2, and Contres are all elongated anteroposteriorly with a brain capsule that is prolate and low (Figs 1 and 3; fig. 4.4 in [11]). Only *D*. *naui* exposes an elongated posterior region of the skull relative to the condylobasal length (Tables 1 and 2). The orbita of both species are generally large in relation to the rest of the skull. The orientation of the orbita, however, is different in both species. In *D*. *naui* the orbita is facing laterally, whereas into *D*. *crassum* the orbita is laterodorsally oriented.

**Table 2:** Cranial differences between males and females of *Dorcatherium naui* and *Dorcatherium crassum*.

Cranial characters	Comparative skull morphology (males and females)	
	*Dorcatherium naui*	*Dorcatherium crassum*
Orientation of orbitae	lateral facing	dorsolateral facing
Postorbital part of skull (Condylobasal length/postfrontal length)	elongated (ratio = 2.9)	shortened (ratio = 4.0)
Separation of supraorbital foramen from supraorbital groove	absent	by a bony bridge
Development of nuchal crests	well-developed	strongly-developed and set off
Occipital crest	present	absent
Development of nuchal tubercle	prominent	Weakly-developed
	**Comparative skull morphology (males)**	
	***Dorcatherium naui***	***Dorcatherium crassum***
Frontal plateau	present (concave)	absent
Frontoparietal bulges	present, well-developed	absent
Morphology of temporal lines	thin and crest-like	Broad and flat
Development of parietal plateau	Strongly-developed, domed posterior skull roof	Almost absent, flat skull roof
Morphology of temporal lines and sagittal crest	temporal lines proceed posteriorly, parallel to the level of the sagittal crest, and merge with the sagittal crest	Temporal lines merge with anterior part of sagittal crest and form a Y-structure

In dorsal view, a W-shaped nasofrontal suture is exposed in both species. In GPIT/MA/17690, the nasofrontal suture is assumed to have the same position as in the specimen from Walda 2 and GPIT/MA/18000-01; however, as GPIT/MA/17690 is broken off at the center of the orbita, the position of the nasofrontal suture is uncertain. At the frontals, supraorbital foramina and the associated deep supraorbital grooves are present in both, *D*. *naui* and *D*. *crassum*. The supraorbital foramina are situated at the level of the anterior orbita margins in all specimens of *D*. *naui*. In *D*. *crassum* from Contres [12] those foramina lie more posteriorly at the posterior margin of the orbita. In contrast, the specimen from Walda 2 shows the same position as *D*. *naui*. As already mentioned by Guzman-Sandoval [11] the position of the supraorbital foramen is highly variable among extant tragulids; similar variations are present in *Dorcatherium*. Only in *D*. *crassum*, a robust bony bridge is separating the supraorbital foramen.

Posterior to the frontoparietal suture, a parietal plateau is only visible in *D*. *naui*, especially in GPIT/MA/17690, but it is almost absent in *D*. *crassum*. Guzman-Sandoval [11] described the skull roof of *D*. *crassum* from Walda 2 as “almost flat surface” resulting in an almost absent parietal plateau. In contrast, in *D*. *naui*, the plateau extends to the posterior end of the skull roof, enclosed by the temporal lines laterally and the medially bending frontoparietal bulges anteriorly. The temporal lines proceed posteriorly to approximate the sagittal crest asymptotic and merge with the posterior part of the latter. In contrast, in *D*. *crassum* from Walda 2 the temporal lines merge at the center of the posterior skull roof and blend into the anterior part of the sagittal crest. Together they form a Y-structure.

In lateral and posterior view, both species expose similar, well-developed nuchal crests. They are more prominent and set off in *D*. *crassum*, especially in NMA-Walda 2 (Figs 4.6 and 4.8 in [11]), than in *D*. *naui*. In all specimens of *D*. *naui*, an occipital crest is present, but this crest is absent in *D*. *crassum* (Figs 2, 6 and 12). Ventrally, the occipital crest merges into well-developed and prominent nuchal tubercles in *D*. *naui*, whereas in *D*. *crassum* the nuchal tubercles are less-developed.

In ventral view, *D*. *crassum* and *D*. *naui* exhibit similar features of the basicranium, in which the latter is slim and elongated with prominent anteroposteriorly elongated anterior tubercles that are divided by a smooth central keel and a large craniopharyngeal foramen ([10]; pl. 2).

Accounting the differences in skull morphology in addition to the well-known differences in dentition (high degree of selenodonty in *D*. *naui* and bunoselenodonty in *D*. *crassum*), the affiliation of *D*. *crassum* to the genus *Dorcatherium*, with *D*. *naui* being the type species of the genus, could be questionable. The evidence provided could be strong enough to support an allocation of *D*. *crassum* into another genus as already suggested by Rössner [2] and Aiglstorfer et al. [8]. However, this has to await future investigations when more fossil tragulid skulls are available.

### Comparison of the known skulls of *Dorcatherium* to extant tragulids

Fossil tragulids, as *D*. *crassum*, have in general a robust morphological appearance as already reported by Guzman-Sandoval [11] and Mennecart et al. [12]. This robustness is characterized by strongly developed neurocranial crests and zygomatic arches, and well-developed sagittal and nuchal crests. The position of the nasofrontal suture is similar in extant tragulids and the known skulls of *Dorcatherium*. The nasofrontal suture of *Dorcatherium* is W-shaped (Fig 4) as in *Tragulus*, but in *Moschiola* ([11]; fig. 4.7) it is V-shaped with the open end pointing anteriorly and in *Hyemoschus* it is almost straight with slight undulations (FMNH 34294).

The supraorbital foramen in extant tragulids is located at the dorsal skull roof at the center of the orbita. The same applies for *Dorcatherium*. The supraorbital grooves are in general larger in *Dorcatherium* and can be separated from the prominent supraorbital grooves by a bony ridge, as visible in *D*. *crassum* [12].

The sagittal crest in *Dorcatherium* is almost in confluence with the nuchal crests as in *Hyemoschus*. However, in *D*. *crassum* and *Hyemoschus* the sagittal crest runs posteroventrally, whereas in *D*. *naui* the sagittal crest and the nuchal crests are at the same level as the parietal plateau (Figs 3 and 8).

*D*. *naui* and *D*. *crassum* show well-developed nuchal crests, much stronger than in *Hyemoschus*. In *Tragulus* and *Moschiola*, the nuchal crests are weak or almost absent [2, 11, 12]. However, in posterior view, the nuchal crests in *D*. *naui* are more slender dorsally than in *D*. *crassum* and *Hyemoschus*, resembling the condition of *Moschiola*.

In posterior view, an occipital crest is present in *D*. *naui* (Figs 6 and 12) and *Hyemoschus* (FMNH 34294) but is absent in *D*. *crassum* (fig 4.8 in [11] and Fig 3 in [12]). Similar to *Hyemoschus*, the dorsal part of the occipital condyles is set off in *Dorcatherium*. The two nuchal tubercles are well-developed and prominent in *D*. *naui*, while they are less-developed in *D*. *crassum* and absent in extant tragulids.

The basicranium of extant tragulids is similar to *Dorcatherium*. They possess a smooth central keel, encompassed by well-developed anteroposteriorly elongated anterior tuberosities and a large craniopharyngeal foramen is visible.

## Discussion

### Intraspecific variability of *Dorcatherium naui*

Both skulls from the Hammerschmiede as well as the skull from Eppelsheim expose differences in the skull roof, in which GPIT/MA/18000-01 possesses delicate temporal lines and sagittal crest, whereas GPIT/MA/17690 and NHMUK PV OR 40632 show prominent frontoparietal bulges and a robust sagittal crest. Both skulls, GPIT/MA/17690 and HMUK PV OR 40632 share similar skulls roof, but the latter additionally possesses large saber-like upper canines (diameter of alveolus 9.2 mm). In contrast, in GPIT/MA/18000-01, only a small alveolus for the upper canine (diameter of alveolus 1.9 mm) is visible. Therefore, GPIT/MA/17690 and NHMUK PV OR 40632 are quite similar in skull morphology (see taxonomic assessment) and differ significantly from GPIT/MA/18000-01 leading to the fact that two morphotypes of *D*. *naui* are present.

#### Interpretation of the different skull morphotypes of *D*. *naui*

As discussed above, there are two different morphotypes in the three skulls of *D*. *naui*. It is evident, that these morphotypes do not represent different species (see systematic Paleontology and taxonomic assessment), but rather represent some kind of intraspecific variation of *D*. *naui*.

It is possible that this intraspecific variation is a result of ontogeny. For juveniles of *Hyemoschus* [12] it is known that the cranial crests are only weakly developed and that they are clearly smaller than the adults. However, the cranial crests in GPIT/MA/17690 and NHMUK PV OR 40632 are very prominent and furthermore these specimens show well-developed frontoparietal bulges. Additionally, all skulls of *D*. *naui* are very similar in size (Table 1) and NHMUK PV OR 40632, as well as GPIT/MA/18000-01 possess adult dentition (Figs 1 and 5). Therefore, it is highly unlikely that the variation found in the two different morphotypes can be explained with juvenile and ontogenetic differences.

**Fig 14:**
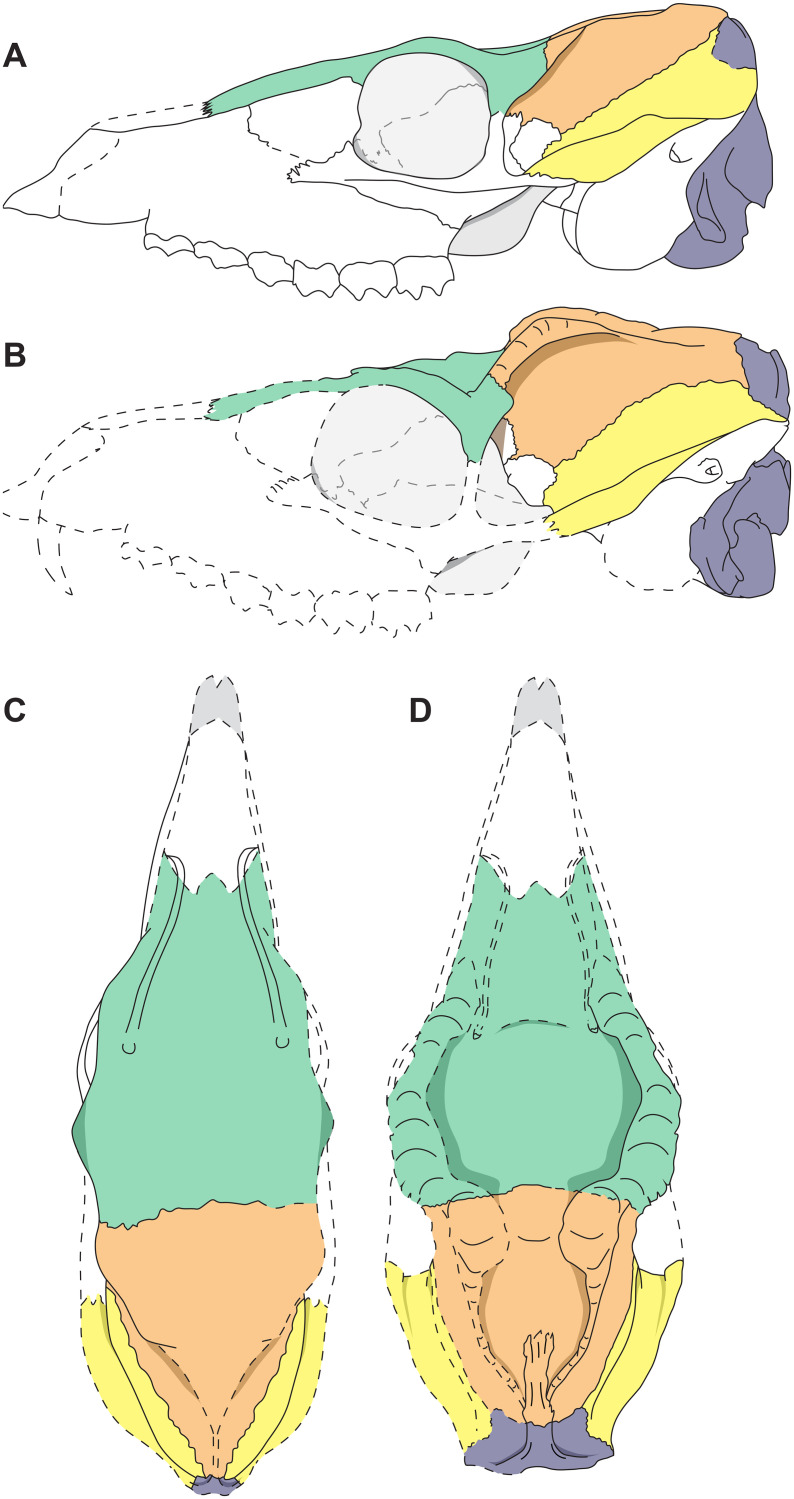
Reconstruction of the male and female skull of *Dorcatherium naui* from the Hammerschmiede locality, Germany. (A) lateral view of GPIT/MA/18000-01 (female), (B) lateral view of GPIT/MA/17690 (male), (C) dorsal view of GPIT/MA/18000-01 (female), (D) dorsal view of GPIT/MA/17690 (male). Green: frontal; Orange: parietal; Yellow: squamosal; Grey: occipital. Skulls not to scale.

The only remaining hypothesis is that both morphotypes are a result of sexual dimorphism as already indicated by the strong difference in size of the upper canine alveoli. Mennecart et al. [12] reported a sexual dimorphism in extant tragulids (*Hyemoschus*) in which the males show stronger-developed cranial crests than females, similar to the skulls of *D*. *naui* described herein, but in general weaker developed. Moreover, male tragulids possess large, saber-like upper canines, which are reduced in females [2]. Based on the canine size, NHMUK PV OR 40632 is a male and GPIT/MA/18000-01 is a female. Because GPIT/MA/17690 is very similar to NHMUK PV OR 40632, although it lacks the anterior part of the snout containing the upper canines, the cranial bulges shared by both specimens indicate that GPIT/MA/17690 is a male and that these bulges are male characters of *D*. *naui* (Fig 14).

#### Sexual dimorphism in *Dorcatherium naui*

Given sexual dimorphism as the most possible hypothesis explaining intraspecific morphological variability in crania of *D*. *naui*, from Hammerschmiede and Eppelsheim, other observed morphological differences could also be related to the same phenomenon of bimodality. Thus, in addition to I) the large saber-like upper canines [2], male skulls of *D naui* are characterized by: (I) a broad, concave and laterally expanding skull roof formed by the anterior frontal (frontal plateau), (II) presence of frontoparietal bulges enclosing a well-developed parietal plateau, (III) a prominent, flat and broad sagittal crest, (IV) large part of occipital participating in the formation of the sagittal crest, (V) strongly-developed nuchal crests, (VI) large and prominent nuchal tubercle, (VII) laterally expanding part of ventral squamosal with only a slight depression, as well as the above mentioned (VIII) large, saber-like upper canines (Fig 14).

In contrast, females exhibit in general a straight and slender outline of the dorsal skull roof (Fig 14A and 14C). Hence, the frontal is smaller, only slightly broadened, and the constriction of the parietal plateau is absent. The sagittal crest is less-prominent and more crest-like (Fig 14C and 14D). Moreover, the most distinctive sexually dimorphic characters are the absence of robust and prominent frontoparietal bulges on the dorsal skull roof (Figs 9 and 14) and the much smaller upper canines (Figs 3 and 5).

### Sexual dimorphism in *Dorcatherium*

#### Sexual dimorphism in *Dorcatherium crissum*

In *D*. *crassum*, large saber-like upper canines are also known from males and therefore, the skull NMA-Walda 2 described by Guzman-Sandoval [11] clearly represents a male. Based on the absence of a large alveolus for the upper canine, the specimen from Thierhaupten NMA-2012-1/2131, described by Seehuber [10], should be a female.

Therefore, the male and the female skull of *D*. *crassum* can be characterized as follows: In dorsal view, the male skull shows broad and flat temporal lines merging posteriorly into a prominent sagittal crest, but in the female skull the gracile temporal lines merge into a more crest-like but narrow sagittal crest. In lateral view, the lateral wall of the braincase of the male skull is posteriorly confined by well-developed nuchal crests, whereas the crests are less prominent in the female.

In the *D*. *crassum* specimen from Contres, the anterior part of the snout is missing and therefore, the canines are not preserved. But based on similarities in the skull morphology such as the broad and flat sagittal crest, the prominent temporal lines, and the dorsally broad nuchal crests that are present in the male specimen NMA-Walda 2 and the *D*. *crassum* specimen from Contres [12] we are confident that both skulls represent males.

#### Comparison of sexual dimorphism in *D*. *crassum* and *D*. *naui*

As discussed above, it is now possible to identify male and female individuals of *Dorcatherium* based not only on the size of the upper canine, but also on characteristics of the skull osteology. In total there are two male skulls and one female skull of both, *D*. *naui* and *D*. *crassum*.

The main difference in skull morphology between both species related to sexual dimorphism is that males of *Dorcatherium* are in general more robust with well-developed cranial crests and females have a more delicate skull morphology with less-pronounced crests and ridges.

**Fig 15:**
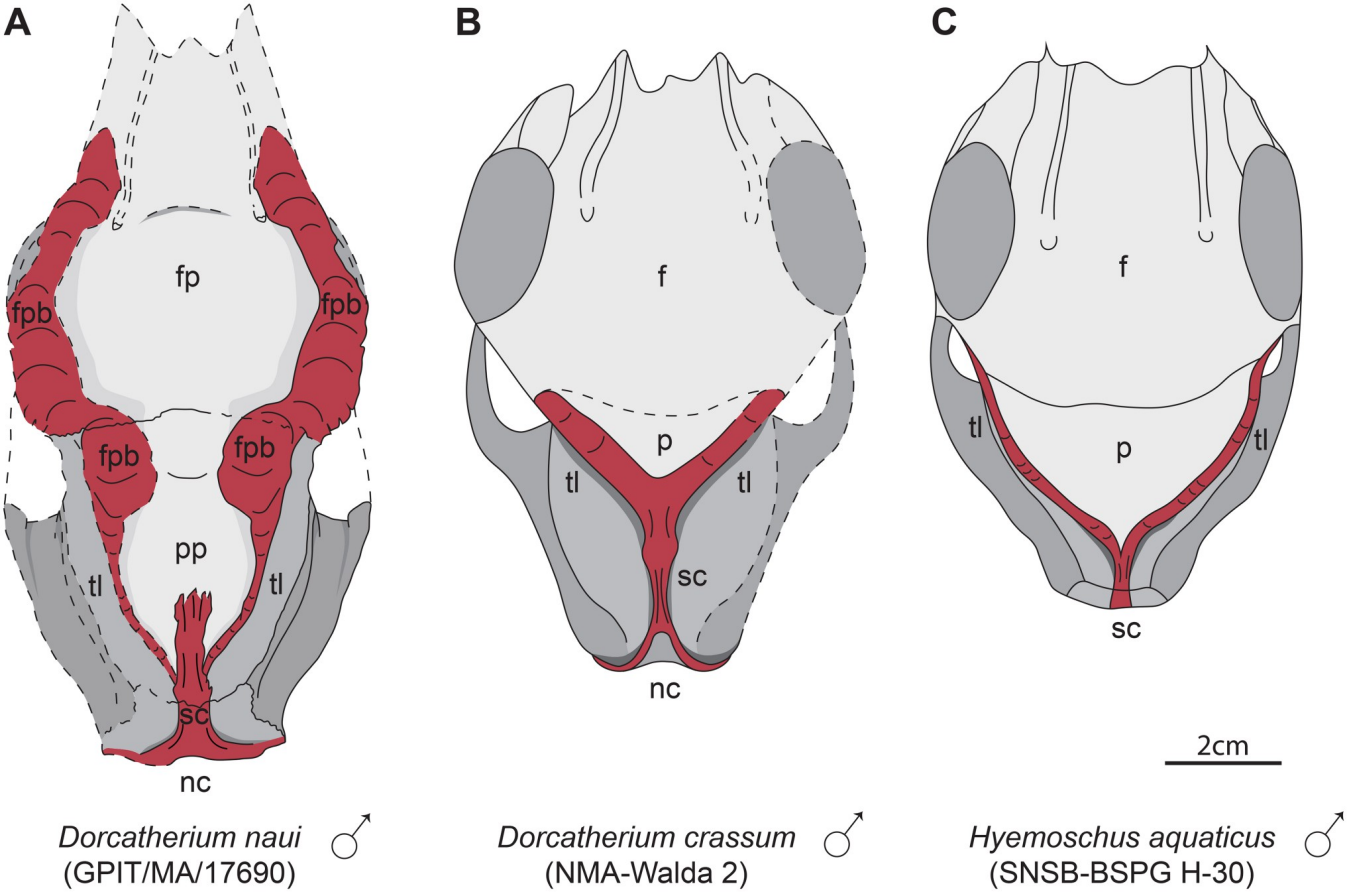
Morphology of the posterior skull roof of males of *Dorcatherium naui*, *D*. *crassum*, and *Hyemoschus aquaticus*. (A) GPIT/MA/17690, *D*. *naui* from Hammerschmiede, Germany (HAM 4), (B) NMA-Walda 2 (drawn after Guzman-Sandoval, 2018), *D*. *crassum* from Walda 2, Germany, (C) SNSB-BSPG H-30 (redrawn after Guzman-Sandoval, 2018), *Hyemoschus aquaticus*. Red: cranial crests and bulges. f, frontal; fp, frontal plateau; fpb, frontoparietal bulges; nc, nuchal crests; p, parietal; pp, parietal plateau; sc, sagittal crest, tl; temporal lines.

In detail, males of both species differ in the presence or absence of lateral bulges at the frontoparietal. In males of *D*. *naui* these bulges, together with the temporal lines enclose the skull roof anteroposteriorly until they merge with the posterior part of the sagittal crest (Figs 14 and 15; Table 2). In males of *D*. *crassum* the frontoparietal bulges are absent, but the temporal lines form two flat ridges, which are only present on the parietals and do not extend onto the frontals.

Males of both species share a broad and flat sagittal crest. The latter is more crest-like and delicate in females of both species. Moreover, males of *D*. *naui* expose a well-developed parietal plateau (Figs 9 and 15), which is only less-pronounced in females of this species (Fig 14). In contrast, in *D*. *crassum*, the parietal plateau is absent in both males and females. Moreover, in *D*. *naui*, the temporal lines proceed posteriorly at the level of the sagittal crest. Here, they run almost parallel to the sagittal crest and merge with the latter slightly anterior to the parietal-occipital suture. In contrast, in males of *D*. *crassum* the temporal lines form a Y-structure (Fig 15).

Moreover, the male skull of *D*. *naui* from the Hammerschmiede contains a concave plateau formed by the frontals anterior to the temporal lines. In contrast, in the male skulls of *D*. *crassum* the anterior skull roof is flat to convex and forms a straight surface extending the posterior part of the skull.

#### Sexual dimorphism in extant tragulids in comparison with *Dorcatherium*

In extant tragulids, sexual dimorphism is long known, but only based on large canines in males and small ones in females. Moreover, Mennecart et al. [12] demonstrated that *D*. *crassum* and *Hyemoschus* share a “hyper-developed” cranial morphology and mentioned that females of *Hyemoschus* show less remarkable characters (e.g. weaker crests), similar to *Dorcatherium* (see above). This is congruent with the herein described features of males and females of *Dorcatherium*, in which males expose well-developed cranial crests (nuchal crests, sagittal crest, and crest-like temporal lines) in contrast to females (Fig 14) and males additionally possess frontoparietal bulges unknown from any other fossil or extant tragulid. Therefore, we agree with Guzman-Sandoval [11] and Mennecart et al. [12] that, based on osteological characters, *D*. *crassum* is more similar to *Hyemoschus* than to *Tragulus* and *Moschiola* ([11]; fig 4.7). As demonstrated above, this is also the case for *D*. *naui*. In detail, the temporal lines of male *D*. *naui* are more similar to *Hyemoschus* than to *D*. *crassum*, but the sagittal crest resembles that of *D*. *crassum*. Males of *D*. *naui* and *Hyemoschus* have similar temporal lines, but the sagittal crest is weaker in extant tragulids and prominent in *Dorcatherium*. In general, males of *Dorcatherium* expose a broad and robust sagittal crest, more developed than in any extant tragulid, whereas in females of *Dorcatherium* the crest is more delicate and less-prominent than in *Hyemoschus*, but somewhat more developed than in *Moschiola* and *Tragulus*.

### Interpretation of the function of the frontoparietal bulges in males of *Dorcatherium naui*

Among land vertebrates, cranial appendages, as well as cranial ornamentation and structures are widely distributed and occur within different vertebrate groups, e.g. [41–47], where they have many purposes and are often used for sexual display or intra-specific combat [48–52]. Pecora e.g. are known for their morphological variety of cranial appendages that resulted in different types of headgear (antlers, horns, pronghorns, and ossicones [53]), similarly used for sexual display and during intra-specific combat [43, 49, 54–57]. Among living ruminants, tragulids are a non-pecoran group, lacking cranial appendages.

Here we can demonstrate that the frontoparietal bulges in males of *D*. *naui* represent a cranial ornamentation previously unknown in extant and fossil tragulids. These bulges are well-developed and markedly set off from the skull roof. The bulges show a rough surface, while the rest of the skull roof has a smooth surface texture (Fig 9) as in some types of headgear known from Pecora (e.g. [53, 58, 59]). Moreover, the results of the μCT-analysis of the male *D*. *naui* from Hammerschmiede revealed that the internal structure is quite similar to bovid horn cores or giraffe ossicones, by being porous or cancellous with surrounding compact bone (Fig 10; compare [41]; fig. 2). Especially, the thickened part of the bulges anterior to the parietal plateau is very porous (Fig 10b cross section 2) and is located on top of the massive and compact frontal bone.

**Fig 16:**
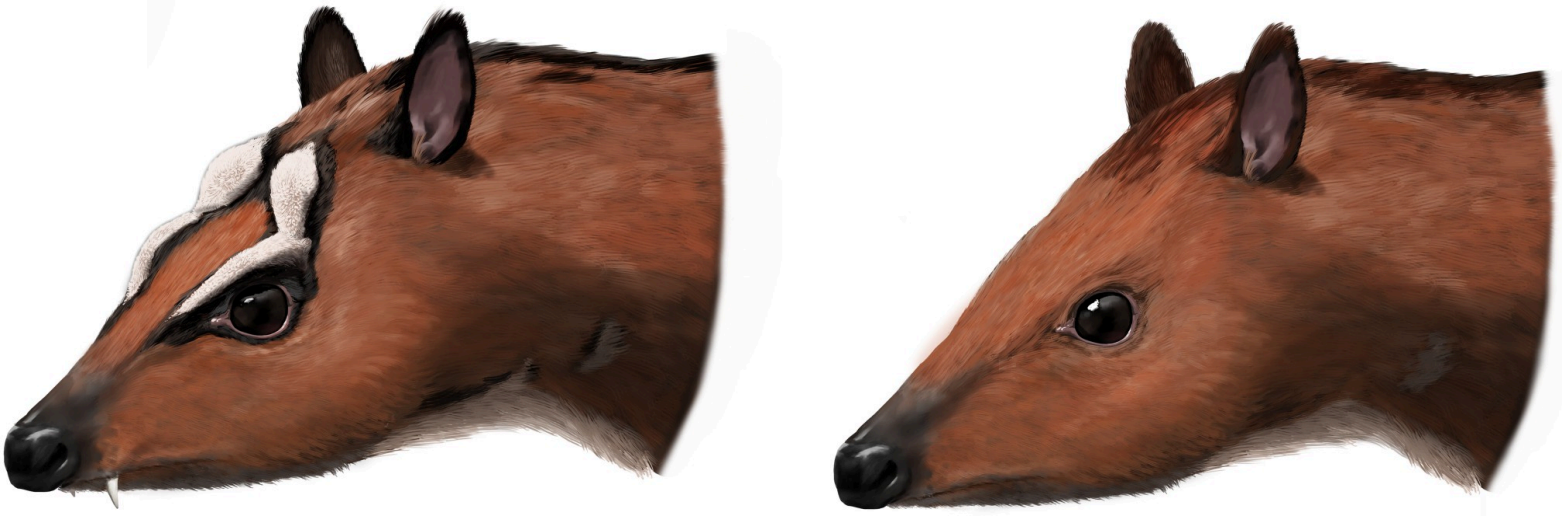
Life reconstruction of male (left) and female (right) *Dorcatherium naui*. Illustration by Peter Nickolaus.

These frontoparietal bulges are only present in males of *D*. *naui* as in Pecora, where cranial appendages are mainly present and stronger developed in males than in females [49, 53, 54, 60, 61]. The bulges in *Dorcatherium* are difficult to interpret, because there is no analogue in tragulids or any other closer related group. However, we here interpret that these bulges were probably used in a similar way as in Pecora—most likely for sexual display (Fig 16).

These bulges, furthermore, cover the dorsal eye-region and could therefore be used in analogy to the frontal bony ridges of extant male *Muntiacus* (Cervidae) which are interpreted to function as a protection against tusk-wounding during male-male combats [62, 63]. The fighting behavior of extant tragulids is dominated by slashing and stabbing with the large upper canines [2, 64] and therefore they develop toughened skin on their backs forming a shield for protection. Hence, an additional protection of the eye region in male *D*. *naui* might be useful.

## Conclusion

For the first time, a detailed description of the skull morphology of the type species of *Dorcatherium*, *D*. *naui*, is provided using three skulls of this species, from the localities of Hammerschmiede (level HAM 4, 11.44 Ma) and Eppelsheim (middle to late Miocene). A comprehensive redescription since Kaups original description in 1839 of the complete skull of *D*. *naui*, NHMUK PV OR 40632 from Eppelsheim, is provided based on a μCT scan. Therefore, an emended diagnosis for *D*. *naui* is presented, which includes for the first time, besides dental features, also characters of the skull such as (I) lateral facing orbitae, (II) prominent nuchal tubercle, and (III) the presence of an occipital crest.

A comparison between the three skulls of *D*. *naui* and the known skulls of *D*. *crassum* shows that these species differ in morphological features of the skull and not only in their dentition. This study revealed that *D*. *naui* differs significantly from *D*. *crassum* in (A) a higher degree of selenodonty, (B) strongly reduced postmetacristid, (C) lateral facing orbitae, (D) separation of supraorbital foramen from supraorbital groove by a bony bridge, (E) well-developed parietal plateau, (F) prominent nuchal tubercle, (G) less-developed nuchal crests, and (H) the presence of an occipital crest. These significant osteological differences between *D*. *naui* and *D*. *crassum*, as well as the close resemblance of other bunoselenodont species with *D*. *crassum* may indicate that they belong to a different genus. This was already suggested by Rössner and Aiglstorfer et al.

This study also showed that unlike other fossil or extant tragulids, *D*. *naui* exhibited significant sexual dimorphism on several cranial features, apart the well-known canine size. The first female skull of this species is described in detail and based on the morphological comparison with the two available male skulls, it is possible to distinguish them by a, more complex and robust skull roof with well-developed crests and bulges in addition to large upper canines in males in contrast to delicate cranial crests and rudimentary upper canines in females. Similar differences between males and the female skull are described here for *D*. *crassum*. Moreover, male skulls of *D*. *naui* differ from males of *D*. *crassum* by the presence of prominent frontoparietal bulges. These bulges were probably used for sexual display and may have functioned as eye protection in male-male combats. In the future, if more skulls of *Dorcatherium* are available, it will be possible to further understand and identify the sexual dimorphism in tragulids.

## Abbreviations

AP: collection A. de Perthuis, Blois, France
FMNH: Field Museum of Natural History, Chicago, USA
GPIT: Geologisch-Paläontologisches Institut Tübingen, Tübingen, Germany
NHMUK: Natural History Museum, London, UK
NMA: Naturmuseum Augsburg, Augsburg, Germany
SNSB-BSPG: Staatliche Naturwissenschaftliche Sammlungen Bayerns-Bayerische Staatssammlung für Paläontologie und Geologie, Munich, Germany
